# The Interplay Between Molybdopterin Synthase and Metal Metabolism Contributes to Pathogenicity in *Acinetobacter baumannii*

**DOI:** 10.1002/advs.78547

**Published:** 2026-10-29

**Authors:** Min Jiao, Wenbo He, Zhenlin Ouyang, Ruochen Wang, Jiaxin Zhang, Xin Liu, Yiting Jing, Qinyue Yu, Xiaoyu He, Qian Qin, Litao Guo, Peter M. Hwang, Fang Zheng, Yurong Wen

**Affiliations:** ^1^ Center For Microbiome Research of Med‐X Institute Department of Critical Care Medicine Shaanxi Provincial Key Laboratory of Sepsis in Critical Care Medicine The First Affiliated Hospital of Xi'an Jiaotong University Xi'an People's Republic of China; ^2^ The Key Laboratory of Environment and Genes Related to Disease of Ministry of Education Health Science Center Xi'an Jiaotong University Xi'an People's Republic of China; ^3^ Longdong University Medical College Qingyang Gansu Province People's Republic of China; ^4^ Departments of Medicine and Biochemistry Faculty of Medicine & Dentistry University of Alberta Edmonton Alberta Canada

**Keywords:** acinetobacter baumannii, bacterial pathogenicity, metal metabolism, molybdopterin synthase

## Abstract

Molybdoenzymes are required in a large amount of essential redox activity and ubiquitously existing in bacteria, archaea, and eukaryotes. The molybdenum cofactor (Moco) is a pterin‐based catalyst, whose synthesis is accomplished by molybdopterin synthase, heterotetrametric MoaE‐MoaD enzyme. We demonstrate that molybdopterin synthase in *Acinetobacter baumannii* plays important roles in bacterial growth, morphology determination, ROS tolerance, biofilm formation, and interplay with the metabolism and homeostasis of other metal ions besides molybdenum. Further, the crystal structure of MoaDE complex was solved and revealed an intrinsically disordered histidine‐rich C‐terminal tail in MoaE that is active in metal chelation and modulating protein stability. *Acinetobacter baumannii* MPT synthase is associated with virulence, which was verified in both *Galleria mellonella* and murine pneumonia model. Our study sheds light on the structural and functional diversity of bacterial molybdopterin synthases and explores crosstalk between different metal homeostasis pathways, providing potential for antibacterial development.

## Graphical abstract

Pathogenic *Acinetobacter baumannii* scavenges metal ions as nutritional elements to survive within the host. This study demonstrates that molybdopterin synthase is essential for pathogenesis and full virulence during infection. Molybdopterin synthase subunit MoaE constitutes an intrinsically disordered C‐terminal histidine rich tail, which mediates metal interaction and homeostasis, uncovering a functional link between molybdenum metabolism and broader metal‐ion homeostasis during host infection.

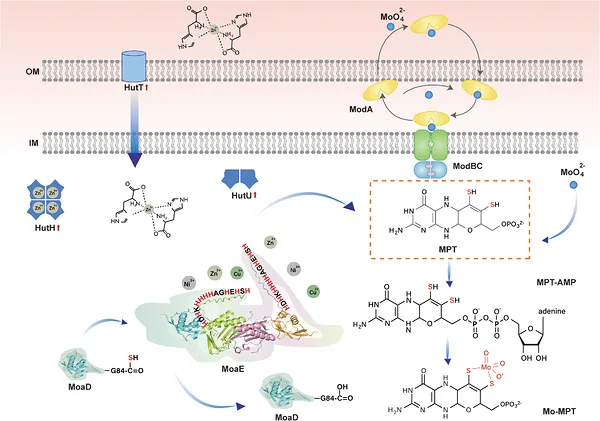

## 1 Introduction

To survive inside a host, an invading pathogenic bacterium must be able to scavenge scarce nutrients, including transition metal elements such as iron, zinc, nickel, and molybdenum [1, 2]. Molybdenum‐containing enzymes, ubiquitously existing in bacteria, archaea, and eukaryotes, with over 50 distinct types of enzymes identified in bacteria, participate in essential redox reactions associated with carbon, nitrogen and sulfur metabolism [3, 4, 5, 6]. Molybdenum is bioavailable as molybdate; bacteria possess the high‐affinity ABC‐type ModABC transporter system for molybdate assimilation [7, 8], whereas eukaryotic MFS‐type molybdate transporters such as MOT1 and MOT2 have also been identified [9, 10]. Once transported into the cell, molybdenum is predominantly bound to the *cis*‐dithiolene group of molybdopterin (MPT) to make the molybdenum cofactor (Moco). The alternative for molybdenum incorporation is the unique iron‐molybdenum cofactor (FeMoco) of bacterial nitrogenase found only in limited species [11, 12, 13, 14, 15]. Moco is unstable and must be newly synthesized in vivo. The synthetic pathway in *Escherichia coli* begins with the formation of cyclic pyranopterin monophosphate (cPMP, precursor Z) from 5’‐ GTP, followed by insertion of two sulfur atoms to generate MPT, which is then adenylated yielding MPT‐AMP, at last covalent incorporation of molybdenum to produce Moco [16]. The biosynthesis is ancient and highly conserved in all organisms including humans [15, 16]. The antimalarial agent artemether targets the Moco biosynthesis cascade [17], and human MPT synthase mutations result in Moco deficiency disease, causing microcephaly and seizures [18, 19].

The second step insertion of a *cis*‐dithiolene group into molybdopterin is catalyzed by MPT synthase, a heterotetrametric enzyme composed of a central MoaE dimer with a MoaD monomer at each side, both encoded by the *moa* operon [20]. The reaction is a dynamic process, with each MoaE monomer binding to a cPMP molecule, and MoaD cycling between multiple different proteins to add two sulfur atoms sequentially to MoaE‐bound cPMP. A sulfur atom from cysteine is transferred by cysteine desulfurase enzyme IscS to TusA, then delivered to the carboxy‐terminal di‐Gly motif of MoaD, previously activated by MoeB. After adding the first thiocarboxylate sulfur to cPMP to form a hemi‐sulfurate intermediate, MoaD dissociates from the MPT synthase complex, then a new MoaD containing an additional thiocarboxylate group (MoaD‐SH) is introduced to the complex, adding the second sulfur atom to complete MPT [16, 21, 22, 23].

MPT synthase assembly and activity can be intricately regulated to optimize environmental adaptation. MPT synthase is usually encoded by two separate genes, *moaD* and *moaE*, within the *moa* operon, while, in pathogenic mycobacteria, *moaX* encodes the MoaD and MoaE domains in a single fused polypeptide, requiring post‐translational cleavage into separate subunits for full MPT synthase activity [24]. A similar fused MPT that is cleaved by JAMM_DR_ was discovered in *Deinococcus radiodurans* [25]. Three homologs of MoaEs and MoaDs were elucidated in *Mycobacterium tuberculosis*, with minor sequence variations showing distinct binding interface features, and structural rearrangements leading to enzymatic inactivation [26]. MPT synthase is strongly associated with ROS tolerance, as *DrmoaE* is upregulated by H_2_O_2_ treatment [25, 27], and defective *DrmoaE* attenuated bacterial growth under such conditions [28]. The presence of fused constructs could illustrate the importance of coordinated expression of the two subunits, which we will test in the current study.

Moco biosynthesis is tightly linked to Fe─S cluster synthesis, consistent with multiple examples of cross‐talk throughout nature between molybdenum and iron [29, 30, 31]. For example, MoaA, the S‐adenosyl‐L‐methionine enzyme responsible for the first step of Moco biosynthesis, is an Fe─S containing protein found in all three kingdoms of life [29, 30, 32]. IscS mobilizes the sulfur atom for Fe─S cluster assembly in Fe─S cluster‐containing proteins, as well as for the dithiolene group of MPT [33]. In addition to Moco, there are also Fe─S cluster or hemes for catalytic subunit in some molybdenum enzyme complex [34]. ModA, a periplasmic molybdate binding protein, also interplays with iron uptake [8]. However, there is currently no evidence for the direct involvement of molybdopterin synthase (MPT synthase) in iron metabolism.

We identified a histidine‐rich C‐terminal tail in MoaE that appears capable of binding transition metals. Metal ions form relatively strong interactions with the imidazole rings in histidine‐rich proteins [35], nearly 80% of which exist in bacteria, presumably for high affinity scavenging purposes. In *Bacillus subtilis*, an (‐XH‐)_n_ histidine‐rich motif in YrpE protein possesses high affinity for binding zinc ions [36]. Pht protein contains an HxxHxH motif that controls Fe, Zn, and Mn homeostasis and bacterial virulence [37]. In *Acinetobacter baumannii*, the non‐canonical histidine‐rich C‐terminal tail of MPT synthase MoaE in metal dependent regulation warrants further investigation.

*Acinetobacter baumannii* is a nosocomial pathogen, with a plastic genome adapted to adversity and stress [38, 39] that allows it to survive antibiotic treatments that typically kill all other bacteria [40]. The World Health Organization (WHO) declared CRAB (carbapenem‐resistant *Acinetobacter baumannii*) as a critical group priority organism, for which novel therapeutics are urgently required [41]. MPT synthase is a potential antibacterial drug target, and MoaE possesses a histidine‐rich terminus tail exclusively in *A. baumannii*. We observed the impact of MPT synthase plus‐or‐minus the poly‐histidine C‐terminal tail of MoaE on bacterial physiology, growth, morphology, ROS tolerance, and biofilm formation. We also performed transcriptome sequencing to further identify the role of MPT synthase in carbon, nitrogen, and sulfur metabolism, distinguishing between MoaD and MoaE, as well as the influence of the polyhistidine tail. We verified interaction of metal ions with MoaE and solved the crystal structure of MPT synthase, demonstrating the flexibility of the histidine‐rich C‐terminal tail of MoaE by SAXS. Finally, the pathogenicity of MPT synthase was studied in infection models of *Galleria mellonella* and mouse acute pneumonia. Our work shows MPT synthase to be a potential target for antimicrobial drug research and development.

## 2 Results

### 2.1 MPT Synthase Impacts Morphology, ROS Tolerance, Biofilm Formation, and Metal Ion Homeostasis in *Acinetobacter baumannii*

The *A. baumannii* ATCC 19606 *moa* operon is comprised of *moaA*, *moaC*, *moaD* and *moaE*, flanked by the *nir* operon related to nitrate metabolism and the *tus* operon associated with tRNA thiolation (Figure 1A, Table S1). MoaD is an 84‐amino acid protein with GenBank accession number WP_001187658.1, MoaE is a 161‐amino acid protein with GenBank accession number WP_000657202.1.

**FIGURE 1:**
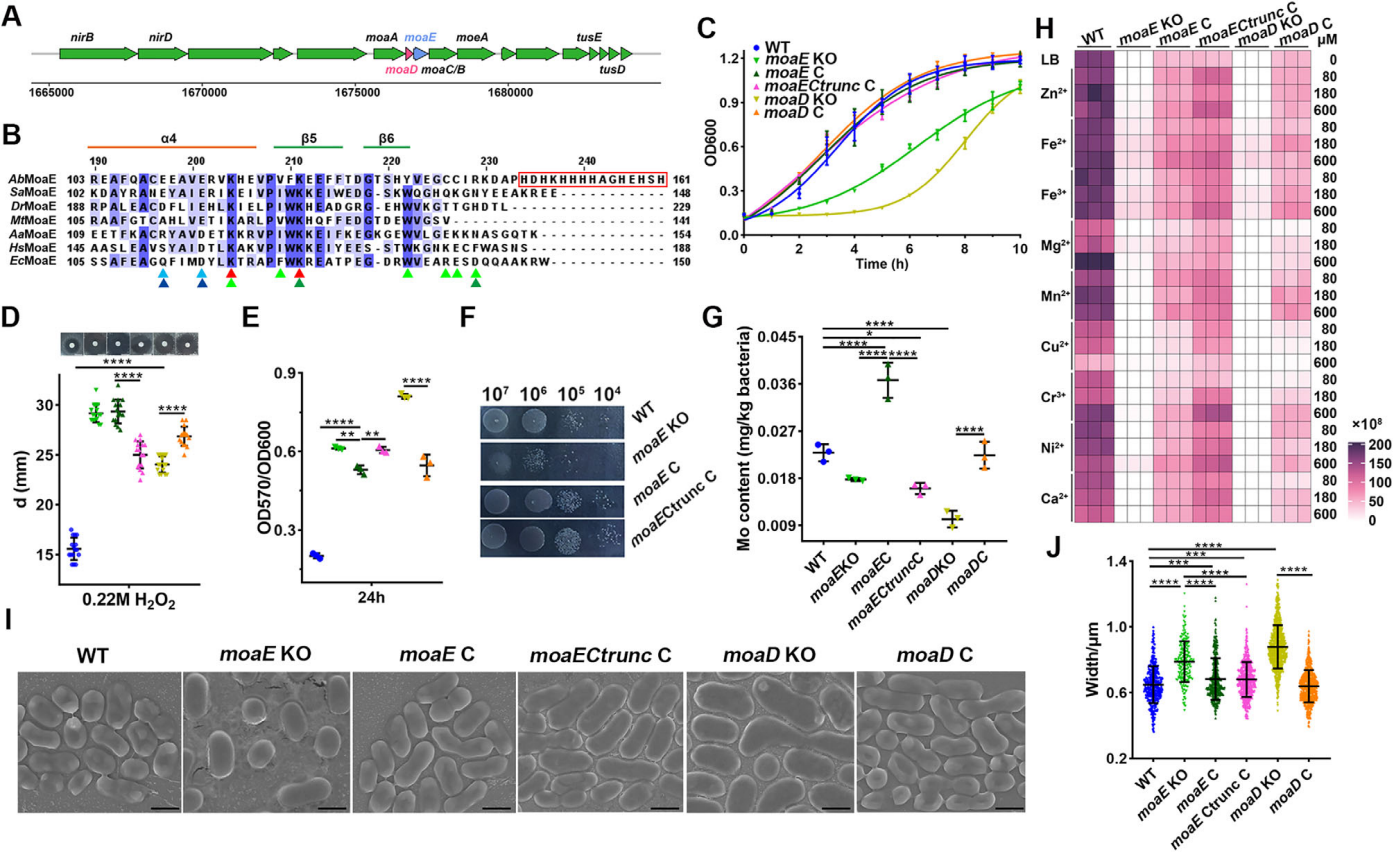
MPT synthase impacts morphology, ROS tolerance, biofilm formation, and metal ion homeostasis in *Acinetobacter baumannii*. (A) Gene map of the *moa* operon in *A. baumannii* ATCC 19606, showing upstream *tus* and downstream *nir*. (B) Alignment of MoaE to top 6 similar MoaEs generated by Dali search shows a unique polyhistidine tail at the carboxyl terminus. Putative cPMP binding amino acids are labeled by red triangles, interaction amino acids between MoaE dimers are marked by blue and deep blue for hydrogen bond and salt bridge, respectively, and interaction amino acids between MoaE and MoaD are indicated by green and deep green for hydrogen bond and salt bridge, respectively. (C) Knockouts of *moaD* (DK) or *moaE* (EK) prolonged bacterial growth lag phase compared to wild type (WT) and complemented *moaD* (DC) or *moaE* (EC) strains cultured in LB medium, with *moaD* knockout strain (DK) shown longer adaptation than *moaE* knockout strain (EK), while *moaE* knockout strain (EK) possessing decreased growth rate during logarithmic phase than *moaD* knockout strain (DK), while, carboxyl terminal truncated *moaE* complemented (ECtrunc) strain grows similarly to WT or EC strain. Different strains were cultured to 10 h with OD600 measurement performed hourly. Values represent means ± standard errors from three independent biological replicates, and WT, EK, EC, ECtrunc, DK and DC are labeled as blue, green, deep green, pink, yellow and orange, respectively. (D) *moaE* knockout (29.16 ± 0.90) or complemented (29.35 ± 1.19) strains significantly increased inhibition zone diameter compared to WT (15.58 ± 1.13), while, carboxyl terminal truncation (25.00 ±1.35) decreased inhibition zone diameter compared to EC complemented strains, still significantly longer than WT. *moaD* knock‐out (24.04 ± 0.81) or complemented (26.86 ± 0.95) strains significantly increased inhibition zone diameter compared to WT (15.58 ± 1.13), with DK's shorter than DC's. *Bars* = 20 mm. Values represent means ± standard errors from three independent biological replicates, and one way ANOVA was performed to calculate the statistical difference in GraphPad Prism, with *P* < 0.0001 marked by four asterisks for EK, EC, ECtrunc, DK or DC compared to WT, EC compared to ECtrunc, and DC compared to DK. (E) *moaD* (0.81 ± 0.01) or *moaE* knock‐out (0.61 ± 0.00), or complemented strains significantly increased the biofilm formation compared to WT (0.20 ± 0.01), with *moaD* (0.55 ± 0.04) or *moaE* complemented (0.53 ± 0.02) strains significantly less than its knockout strains, respectively. While, carboxyl terminal truncation of EC (0.61 ± 0.01) shows no difference to EK (0.61 ± 0.00), which is significantly higher than EC. Values represent means ± standard errors from three independent biological replicates, and one way ANOVA was performed to calculate the statistical difference in GraphPad Prism, with *P* < 0.0001 labeled by four asterisks for EC compared to WT, DC compared to DK, *p* = 0.0027 labeled by two asterisks for EC compared to EK, *p* = 0.0053 labeled by two asterisks for ECtrunc compared to EC. (F) Similar growth pattern of *moaE* gene knock‐out or complemented strains to LB medium were detected by 10‐fold serial dilution and spot plated on M9 minimal medium nearly without molybdate agar plate. (G) The content of molybdenum. All statistics significances were calculated by one‐way ANOVA via GraphPad Prism, with four asterisks indicating *p* value < 0.0001, and one asterisk for *p* value < 0.05. (H) Heatmap of bacterial CFU in response to different metal ions. EC, DC and ECtrunc show similar growth pattern compared to WT adapting to different kind and content metal ions, with ECtrunc showing little higher density than EC. DK and EK strains barely grow, with Fe^2+^ and Fe^3+^ recovering some extent survival especially over 180 µm, 600 µm Zn^2+^, 180 µM Mg^2+^ and 600 µm Ni^2+^ surviving some extent EK instead of DK. Three independent biological replicates were performed, with the density of CFU from 0 to 200 (10^8^ ×) marked from pink to purple. (I) *moaD* or *moaE* knock‐out strains show abnormal morphology with larger size and decreased cell adhesion compared to WT, *moaD* or *moaE* complemented strains, while complemented with *moaECtrunc* can recover morphology to some extent but still abnormal under scanning electron microscope (SEM) observation. Images are selectively shown after three independent biological replicates with *Bars* = 1 µm. (J) Statistical analysis of cell width shows significant increase after *moaD* or *moaE* knock‐out. Complemented with *moaD* or *moaE* can reverse this phenomenon, while the width of *moaECtrunc* complementation strain is shorter than *moaE* complementation strain. Four asterisks represent *p*<0.0001, calculated by one‐way ANOVA in GraphPad Prism with three independent biological replicates.

Sequence similarity networks were constructed to describe the evolutionary relationships of MoaD and MoaE. First, PSI‐BLAST searches were performed and generated 713 and 1400 similar protein homologs with over 40% sequence identity to MoaD and MoaE, using non‐redundant protein and metagenomic bacterial protein databases, including 10 orders of bacteria from the compost metagenome, marine metagenome, and unclassified other species. Then homologs were collected, 27 and 400 sequences corresponding to MoaD and MoaE, respectively, for further clustered sequence similarity network exhibition with 100% edge percentage identity with *Acinetobacter* homologs cutoff. MoaE is relatively conserved as located near to the center with 79798 edges, while MoaD showed specific to others as located at edge with 318 edges, consistently 96.2% homologs to MoaD were from *Acinetobacter*, while 71.4% orthologs to MoaE belong to *Acinetobacter* (Figure S1A).

The protein alignment of MoaE with the top 6 similar structures displayed a noncanonical C‐terminal histidine‐rich sequence: “HxHxHHHHxxHxHxH” (Figures 1B, S1B), and this kind of similar carboxyl terminal tail of MoaE is widely present in *Acinetobacter*, with the median number of histidine residues of six compared to other bacteria genera (Figures S1C, S1D). Histidine‐rich proteins play vital roles in extracting metal elements for bacteria infecting hosts [1, 35]. We designed an MoaE C‐terminally truncated strain (ECtrunc), as well as *moaD* and *moaE* knockout and complemented strains, which the gene expression has been verified by qRT‐PCR (Figure S2A), for our bacterial physiology studies (Table S2).

Cultured in LB medium, knockouts of *moaD* (DK) or *moaE* (EK) showed significant attenuation of growth compared to *A. baumannii* ATCC 19606 wild type (WT), *moaD* (DC), or *moaE* (EC) complemented strains, with the absence of *moaD* (DK) leading to a much longer lag phase than *moaE* knockout (EK). Knockout of *moaE* (EK) also decreased the growth rate in logarithmic phase. The *moaE* ECtrunc strain grew similarly to *A. baumannii* ATCC 19606 wild type (WT) and *moaE* complemented (EC) strain (Figure 1C). Intact MPT synthase is important for H_2_O_2_ tolerance, with *moaD* (DK) or *moaE* (EK) knockout having decreased resistance to H_2_O_2_. Notably, complementation (DC, EC or ECtrunc) strains did not restore H_2_O_2_ resistance, possibly due to dyscoordination of MoaD and MoaE in Moco biosynthesis (Figure S2A). MoaE C‐terminal truncation also decreased sensitivity to H_2_O_2_ compared to MoaE (Figure 1D). Biofilm formation increased after MPT synthase knockout compared to *A. baumannii* ATCC 19606 wild type (WT), with a persistent but lesser effect observed in the *moaD* (DC) and *moaE* (EC) complementation strains. Biofilm formation in the *moaE* C‐ terminal truncation complementation (ECtrunc) strain was similar to the *moaE* knockout (EK) strain (Figure 1E). Consistent growth phenotype was observed in barely detectable molybdate M9 medium (Figure 1F) compared to LB medium (Figure 1C). Intracellular molybdenum content has been used as an indirect indicator of altered molybdenum metabolism. Decreased intracellular molybdenum content was detected after *moaD* or *moaE* knock‐out compared to *A. baumannii* ATCC 19606 wildtype (WT), *moaD* or *moaE* complementation recovered the molybdenum content, while C‐terminal truncation of *moaE* failed to recover (Figure 1G).

We further studied the impact of metal ion supplementation on bacterial growth. *moaD* (DC), *moaE* (EC), MoaE C‐terminal truncation (ECtrunc) complemented strains and WT showed similar growth patterns under different metal ion concentrations, while the CFU of MoaE C‐terminal truncation complemented (ECtrunc) strain is higher than MoaE complemented (EC) strain. Most strikingly, addition of Fe^2+^ or Fe^3+^ can partially rescue growth of *moaD* (DK) and *moaE* (EK) knock‐out strains, whereas Zn^2+^, Mg^2+^ and Ni^2+^ have a more modest effect for the *moaE* (EK) knockout strain (Figure 1H). Scanning electron microscopy (SEM) images demonstrated abnormal morphology with less adhesion between cells and enlarged size after *moaD* or *moaE* knockout (Figure 1I), with significantly greater width, compared to wildtype (WT), *moaD*, or *moaE* complemented strains (Figure 1J). MoaE C‐terminal truncation (ECtrunc) strain had slightly greater width, but exhibited less of change than the knockout strains (Figure 1I,J).

### 2.2 MPT Synthase Is Involved in Elemental Metabolism and Metal Ion Homeostasis, Mediating Bacterial Pathogenicity

RNAseq of *moaD* (Figure 2A) or *moaE* (Figure 2B) knock‐out strains compared to wild type and complemented strains were conducted to uncover interaction networks for MPT synthase. Metal transportation was disrupted by MPT knock‐out, *modA*, *merR*, and *cueR* were up‐regulated after *moaD* (Figure 2A) or *moaE* (Figure 2B) knock‐out, while *nikR* (13835) [42] was down‐regulated and further confirmed by qRT‐PCR (Figure S2). For accuracy evaluation, DEGs intersection was performed for *moaD* (Figure 2D) and *moaE* (Figure 2F), respectively. WT or complemented strains live like normal resident flora, building cell envelopes and communicating with each other, with the following genes showing increased expression: *metF*, encoding a step in methionine biosynthesis [43], glycosyltransferase in cell envelope biogenesis [44], *tolB* or *15620* (*tolQ*) in Tol biopolymer transport system of T1SS [45], and signal transduction related nitrogen regulation protein NR(I), *benA* and *paaC* of the aromatic metabolism pathway [46, 47], as well as *katE* involving metal ion transport and ROS tolerance [48] (Figure 2A,B), which were validated by qRT‐PCR (Figure S2). Knockout of *moaE* (Figure 2B,D) showed more widespread influence than *moaD* (Figure 2A,F), with 41 versus 16 DEGs, respectively, with over 2.5 times numbers of down‐regulated DEGs being detected, including genes *06835* (*wzb*), *06840* (*wza*) [49, 50], and 08815 (*lgt*) associated with envelope biogenesis, and *GGDEF* domain‐containing protein for signal transduction. There are also genes *betI* and *betB* related to osmotic stress [51], and further validated by qRT‐PCR (Figure S2), as well as *barB* and *basG* for iron homeostasis [52] (Figure 2E). On the other hand, after *moaE* (Figure 2B,F) or *moaD* (Figure 2A,D) knockout cells are depleted and virulent, with 111 or 69 DEGs showing increased expression, biofilm formation related PEGA domain‐containing protein involved in the synthesis and export of poly‐β‐(1,6)‐N‐acetylglucosamine [53], transporters including amino acid permease, sulfate ABC transporter and MFS transporter, weapons like *00550* (GIY‐YIG) [54, 55] or 00670 (VRR‐NUC) [56] domain containing nuclease for physical contacting inhibition, together with their assistant *tssM* or PAAR domain containing proteins of T6SS [45, 57]. There are also up‐regulated DEGs specific in *moaE* mutant strain, such as histidine utilization *hut* operon genes *hutH*, *hutU* associated with nitrogen and zinc homeostasis [58]. As well as *ahpF* hydroperoxide reductase in *moaD*‐lacking strain [59] (Figure 2E).

**FIGURE 2:**
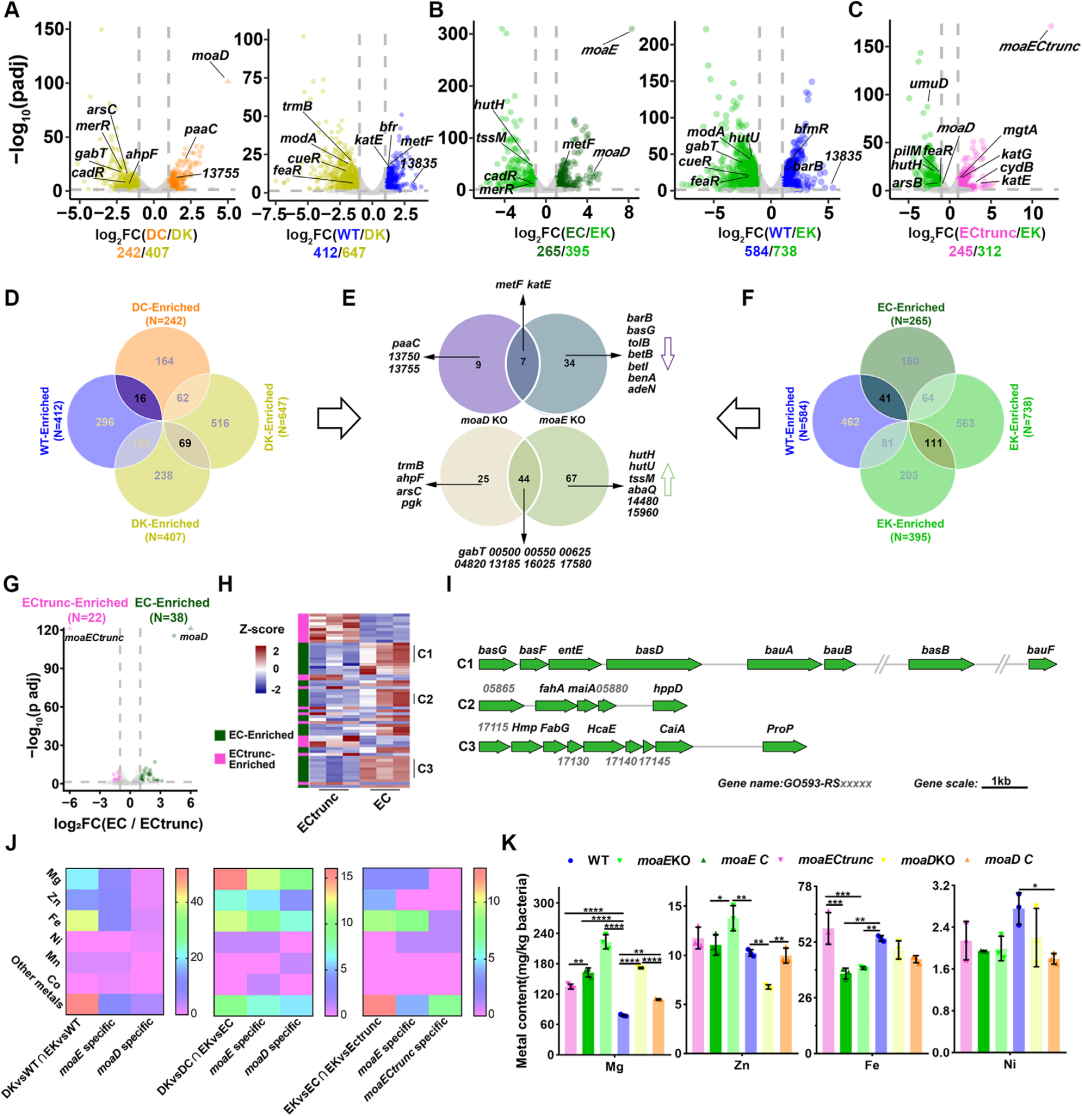
MPT synthase is involved in elemental metabolism and metal ion homeostasis, mediating bacterial pathogenicity. (A) Volcano plot of differentially expressed genes (DEGs) in DK strain versus WT and DC by RNA‐sequencing. Significant DEGs were filtered by adjust p value (p‐adj) < 0.05, and |log2 fold change (FC)|>1. There was total 407 significantly down‐regulated DEGs and 242 significantly up‐regulated DEGs were detected in DC versus DK, while, 647 significantly down‐regulated DEGs and 412 significantly up‐regulated DEGs in WT group compared to DK. *13835*(*nikR*), *katE*, *paaC*, *metF*, and *13755* were significantly down‐regulated, while *modA*, *feaR*, *cadR*, *arsC* and *gabT* show up‐regulated after *moaD* knock‐out. X axis markers log2 FC, Y axis markers ‐log10 (p adj). (B) Volcano plot of DEGs in EK strain versus WT and EC by RNA‐sequencing. Significant DEGs were selected by adjust p value (p adj) < 0.05, and |log2 fold change (FC)|>1. Total 395 significantly down‐regulated DEGs and 265 significantly up‐regulated DEGs were screened out in EC versus EK, (E) while, 738 significantly down‐regulated DEGs and 584 significantly up‐regulated DEGs were identified in WT group compared to EK. *13835*(*nikR*), *barB*, *bfmR*, and *metF* were significantly down‐regulated, while *modA, hutH*, *hutU*, *cadR*, and *merR* show up‐regulated after *moaE* knock‐out. X axis represents log2 FC, Y axis represents ‐log10 (p adj). (C) Volcano plot of DEGs in ECtrunc strain versus EK by RNA‐sequencing. Significant DEGs were selected by adjust p value (p adj) < 0.05, and |log2 fold change (FC)|>1. Total 245 up‐regulated genes including *moaECtrunc*, *mgtA* and 312 down‐regulated genes including *moaD*, *hutH* were identified. X axis represents log2 FC, Y axis represents ‐log10 (p adj). (D) Venn diagram generated from DEGs illustrates that there are 16 up‐regulated and 69 down‐regulated DEGs common to both wild type (WT) and *moaD* complementation (DC) strains compared to the *moaD* knockout (DK) strain. (E) Venn diagram generated from expression pattern of *moaD* or *moaE* deficiency. There are 7 down‐regulated DEGs and 44 up‐regulated DEGs common to both *moaD* and *moaE* knockout strain. (F) Venn diagram generated from DEGs illustrates that there are 41 up‐regulated and 111 down‐regulated DEGs common to both wild type (WT) and *moaE* complementation (EC) strains compared to *moaE* knockout (EK) strain. (G) Volcano plot of DEGs in ECtrunc strain versus EC by RNA‐sequencing. Significant DEGs were selected by adjust p value (p adj) < 0.05, and |log2 fold change (FC)|>1. Total 22 up‐regulated genes including moaECtrunc and 38 down‐regulated genes including *moaD* were identified. X axis represents log2 FC, Y axis represents ‐log10 (p adj). (H) Heatmap of DEGs of ECtrunc versus EC by hierarchical clustering. The increasing expression level based on Z score was displayed by colors from blue to red. (I) Three clusters (labeled by C) were marked for containing at least 5 genes in one operon. Up and down regulated DEGs with ECtrunc compared to EC are indicated by pink and green, respectively. (J) Heatmap of the number of DEGs related to metal. Magnesium (Mg), zinc (Zn), iron (Fe), nickel (Ni), manganese (Mn), cobalt (Co), and metal, were counted in the excel, generated by intersections of DK and EK compared to WT, DK compared to DC and EK compared to EC, EC and ECtrunc compared to EK. (K) The content of magnesium, zinc, iron and nickel. All statistics significances were calculated by one‐way ANOVA via GraphPad Prism, with four asterisks indicating *p* value < 0.0001, three asterisks for *p* value < 0.001, two asterisks for *p* value < 0.01, and one asterisk for *p* value < 0.05.

C‐terminal poly‐histidine tail truncation of MoaE was also performed RNAseq, DEGs were generated by comparing ECtrunc to EK (Figure 2C) or EC complemented strain (Figure 2G), respectively. After truncation of histidine tail, the change is relatively slight with 22 up‐regulated and 38 down‐regulated DEGs detected (Figure 2G). Clustered by gene locus (Figure 2H), aromatic catabolism including phenylalanine degradation and iron homeostasis were the main affected pathways when considering the numbers of DEGs and their related functions, like aromatic ring hydroxylating dioxygenase and *miaA* of phenylalanine metabolic pathway [60], as well as iron homeostasis *ent*, *bas*, and *bau* genes responsible for iron assimilation [61] (Figure 2I), which were further validated by qRT‐PCR (Figure S2).

To confirm the involvement of MPT synthase in metal homeostasis, the numbers of DEGs related to different metals were harvested. The most abundant DEGs related to metal ions were identified in gene knock‐out strain, when compared to wildtype (Figure 2J). Besides other metals, the numbers of magnesium, zinc, and iron related DEGs showed significant changes between *moaE* and *moaD* (Figure 2J), while by C‐terminal histidine‐rich truncation, the number of magnesium and iron related DEGs are changing (Figure 2J), the consistent metal content changing was detected in different strains by ICP‐MS, as *moaE* knock‐out strain showed the greatest change in magnesium, zinc, and iron, compared to wildtype, magnesium and iron showed significant change after the truncation of C‐terminal histidine‐rich tail compared to *moaE* complemented strain (Figure 2K).

### 2.3 The MPT Synthase Structure Exhibits an Intrinsically Disordered C‐terminal Tail

Individually overexpressed using pET28a, MoaD eluted as a monomer, whereas MoaE eluted as a dimer on size exclusion chromatography (Figure S3A,B). ITC experiments show an interaction ratio of 2:2 for MoaD to MoaE, with a dissociation constant of about 939 nM (Figure 3A), nearly eighteen times higher affinity than inactive MPT synthase in *Escherichia coli* [62]. The crystal structures of MPT synthase from *A. baumannii* were determined at 2.0 Å, and the X‐ray diffraction data collection and relevant statistics are summarized in Table S3. The structural model includes residues 3–84 of MoaD (full length, 1–84 residues) and 1–143 of MoaE (full length, 1–161 residues). The MPT synthase shows the expected central MoaE dimer, flanked on either side by an MoaD subunit, in which the carboxyl‐terminal di‐Gly motif is strategically inserted into the adjacent MoaE subunit (Figure 3B), where it could potentially donate a sulfur atom to form the dithiolene group at the molybdenum binding site. MoaD consists of four β‐strands (β1, β2, β3, β4) and three α‐helices (α1, α2, α3) located on each side. MoaE consists of three β‐strands (β1, β3, β4) surrounded by two α‐helices (α1, α2) on one side and two α‐helices (α3, α4) on another side, along with an additional four β‐strands (β2, β5, β6, β7). The active site of MPT synthase is located at the junction of the MoaE dimer and the MoaD C‐terminus, and surrounded by conserved Lys, Arg, and His residues [63] (Figure S1B). The structure of *A. baumannii* MPT synthase can be seen binding seven sulfate ions from the crystallization solution (Figure 3B).

**FIGURE 3:**
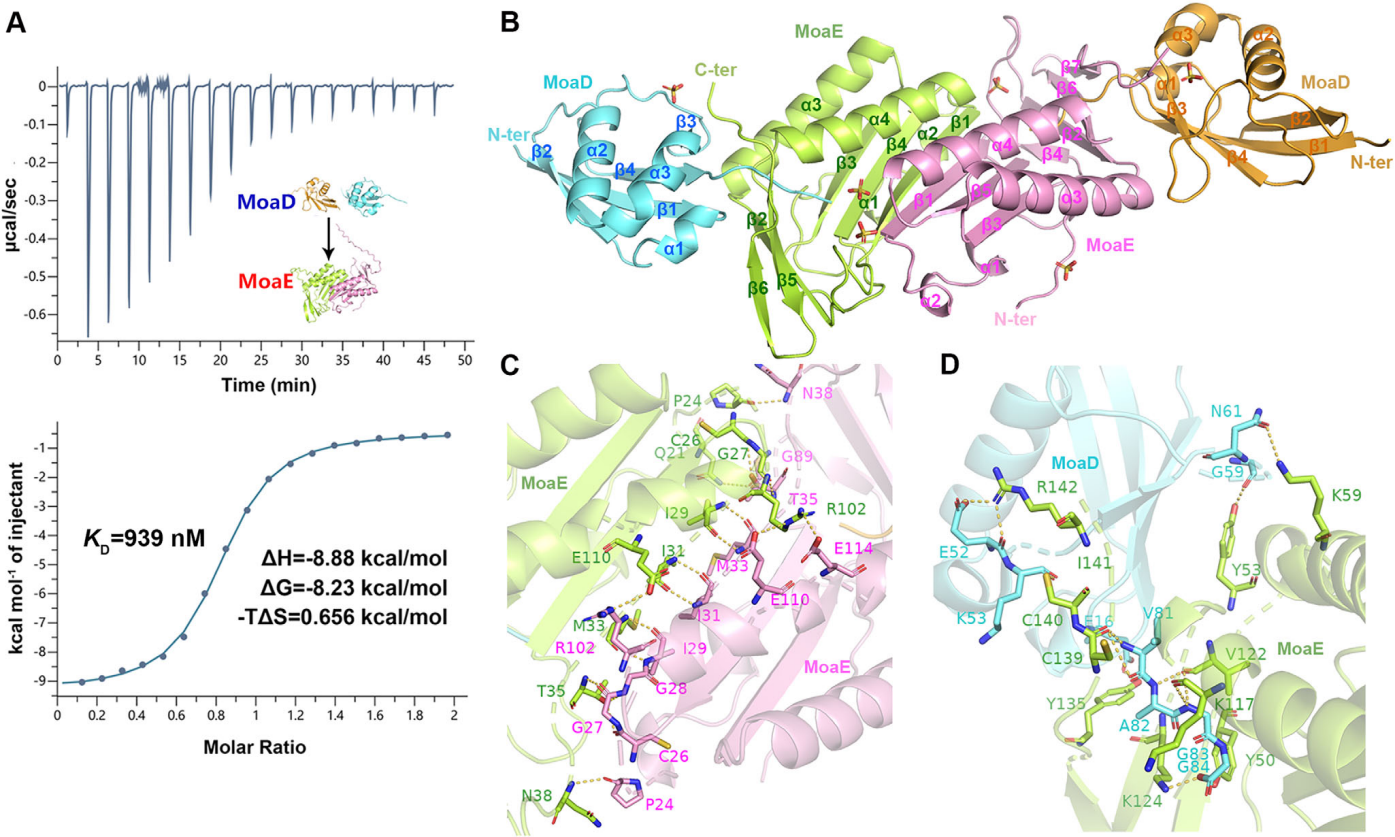
Structural insight in MPT synthase reveals MoaE exhibits an intrinsically disordered C‐terminal tail. (A) Isothermal titration calorimetry (ITC) curves of MoaD against MoaE. The typical data was exhibited with three repeats performed. (B) Overall crystal structure of *Apo*‐MPT synthases inactivity state in ribbon diagram. MoaE dimers in the central are colored limon and pink, respectively, flanking MoaDs are colored aquamarine and brightorange, respectively. N‐ter or C‐ter represents amino terminal or carboxyl terminal, respectively. (C) Details of the interaction interfaces between MoaE dimers, the residues involved in the interaction of N38, T35, M33, I31, I29, Q21, R102, P24, C26, G27, E110 from one MoaE (limon) and P24, C26, G27, I29, I31, M33, G89, E110, E114, N38, T35, M33, G28, R102 from another MoaE (pink). Hydrogen bonds or salt bridges are shown as orange dashed lines, and details are listed in Table S6. (D) Details of the interaction interfaces between MoaD (aquamarine) and MoaE (limon), the residues involved in the interaction of N61, N52, K53, G59, N61, V81, G84, V81, A82 and G83 from MoaD and Y135, R142, C140, Y53, K59, C139, Y50, K124, V122, K117 and V122 from MoaE. Hydrogen bonds or salt bridges are shown as orange dashed lines, and details are listed in Table S7.

MoaE dimerizes via a central six‐stranded β‐sheet, with the interface between MoaEs formed by antiparallel β1 and β1^*^ strands (Figure 3C, Figure S1B and Table S4). Each MoaD interacts with one MoaE and two (MoaE‐MoaD) heterodimers are structurally identical. The interface amino acids mainly from the C‐terminal of MoaD including the canonical di‐Gly and the carboxyl terminal of MoaE close to noncanonical polyhisdines tail (Figure 3D and Table S5). Top 6 similar structures for MoaD and MoaE were generated by Dali search (Table S6), sequence alignment of MoaDs (Figure S3C) or MoaEs (Figure S1B) displayed 20.4% and 30.2% similarity, respectively, and structural surperposition of MoaDs (Figure S3D) and alignment of MoaEs (Figure S3E) indicated conserved interfaces between all of the subunits.

### 2.4 The Histidine‐Rich Carboxyl Terminal of MoaE is Disordered, and Required for Protein Stability Especially Upon the Binding of Metal Ions

The Kratky Plot generated by small angle scattering (SAXS) assay demonstrates that the C‐terminal histidine‐rich tail of MoaE is intrinsically disordered, as further evidenced by its invisibility in the X‐ray crystal structure and flexibility with 20 different models generated by AlphaFold 3 (Figure 1B, Figure 4A, Figure S1B, Figure 3B, Figure S3F). The chi‐squared fitting to SAXS data dropped significantly from 50 to 1.4 by modeling a flexible histidine‐rich carboxyl terminal of MoaE to the solved complex structure of MPT synthase (Figure 4B).

**FIGURE 4:**
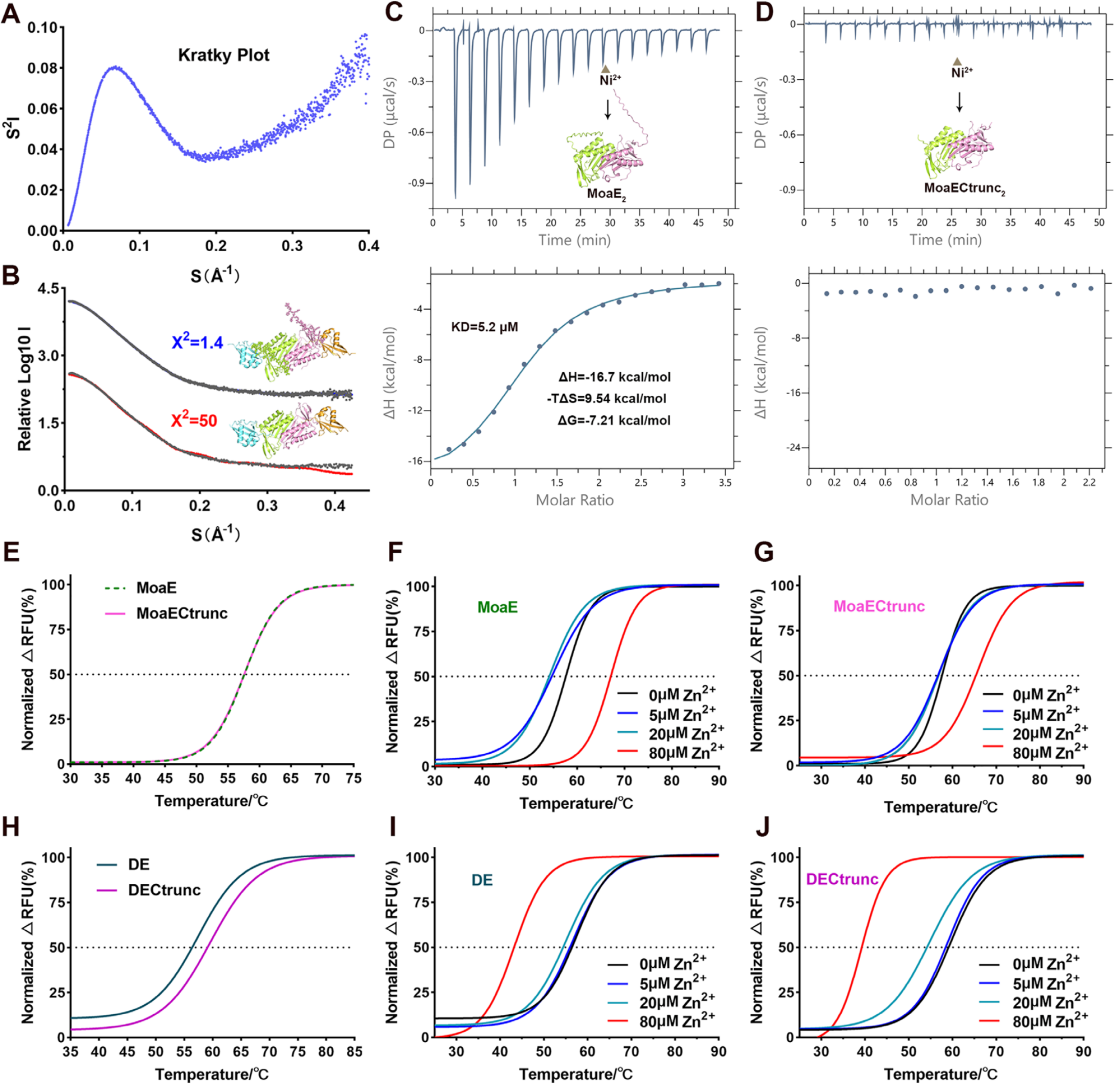
Histidine‐rich carboxyl terminal of MoaE is responsible for protein stability and metal binding. (A) The Kratky plot of MPT synthases in solution by small angle X‐ray scattering indicates a partially intrinsically disordered protein complex. (B) Small angle X‐ray scattering experiment scattering curve of MPT synthases in solution and their fit to the crystal structure of full length (blue top) by CORAL and histidine‐rich carboxyl terminal missed (red bottom) by DAMMIF. The limon and pink spheres represent the missing poly histidine carboxyl terminal tail of two MoaEs in the center, respectively. (C) Isothermal titration calorimetry experiment indicates the thermodynamics of the interaction of MoaE with Ni^2+^. (D) The truncation of histidine‐rich carboxyl terminal of MoaE abolish its interaction with Ni^2+^ detected by isothermal titration calorimetry. (E) The truncation of histidine‐rich carboxyl terminal of MoaE doesn't change the *T_m_* of MoaE dimer by heat shift assay. (F) Heat shift assay shows 80 µm Zn^2+^ increases the *T_m_* of MoaE dimer, while 5 or 20 µm Zn^2+^ decreases the *T_m_*. (G) Heat shift assay shows 80 µm Zn^2+^ increases the *T_m_* of MoaE dimer with carboxyl terminal truncated, while 5 or 20 µm slightly decreases the *T_m_*, while the extent is less than intact MoaE. (H) The truncation of histidine‐rich carboxyl terminal of MoaE increases the *T_m_* of MPT synthase by heat shift assay. (I) Heat shift assay shows 80 µm Zn^2+^ decreases the *T_m_* of MPT synthase, and 20 µM Zn^2+^ slightly decreases the *T_m_*, too. (J) Heat shift assay shows 80 µm Zn^2+^ decreases the *T_m_* of MPT synthase with MoaE histidine‐rich carboxyl terminal truncated, and 20 µm Zn^2+^ decreases the *T_m_*, too, and the extent is more than intact MoaE.

Given the potential metal ion‐binding capacity of the histidine‐rich C‐terminal tail, we performed an ITC interaction assay between MoaE with metal ions, indicating that Ni^2+^ (Figure 4C), Cu^2+^ (Figure S4A) and Zn^2+^ (Figure S4C) can bind to MoaE with affinity of 5.2, 3.4, and 240 µm, respectively, while no visible interaction was detected with Fe^2+^ (Figure S5A), Fe^3+^ (Figure S5B), Mg^2+^ (Figure S5C), Mn^2+^ (Figure S5D), Cr^3+^ (Figure S5E) or Ca^2+^ (Figure S5F). The interactions with Ni^2+^ (Figure 4D), Cu^2+^ (Figure S4B) and Zn^2+^ (Figure S4D) were abolished by carboxyl terminal truncation of MoaE.

The truncation of carboxyl terminal didn't affect the *T_m_* of MoaE dimer during thermal shift assay (Figure 4E), while modulated its sensitivity to Zn^2+^ (Figure 4F,G), Ni^2+^ (Figure S4E,F) and Cu^2+^ (Figure S4G,H). 5 or 20 µm Zn^2+^ (Figure 4F,G) or Ni^2+^ (Figure S4E,F) reduced the *T_m_*, while 80 µm Zn^2+^ elevated the *T_m_* (Figure 4F,G), the magnitude change of MoaE dimer is more significantly than C‐terminal truncated MoaE. For MPT synthase, the *T_m_* was increased by the truncation of carboxyl terminal of MoaE (Figure 4H), but decreased by Zn^2+^ (Figure 4I,J) and Cu^2+^ (Figure S4K,L), except Ni^2+^ (Figure S4I,J). 20 or 80 µM Zn^2+^ decreased the *T_m_* of MPT synthase (Figure 4I) with stronger magnitude change by C‐terminal truncation of MoaE (Figure 4J). While, the C‐terminal truncation of MoaE (Figure S4L) reduced the extent of *T_m_* change of MPT synthase (Figure S4K) caused by 80 µM Cu^2+^. Coincidentally, copper bound to the molybdopterin dithiolene sulphur intermediate was observed in *Arabidopsis thaliana* [64].

### 2.5 MPT Synthase is Associated with Virulence Attenuation When Disrupted in the Infection of *Galleria mellonella* or mouse by *A. baumannii* ATCC 19606

Metabolic defects caused by disrupted MPT synthase may exert indirect effects on bacterial virulence. The infection models of *G. mellonella* or murine pneumonia with cyclophosphamide‐induced neutropenia were conducted for lethality and lung invasion assay, respectively. Survival curves were recorded for wax larvae after injection at the last left proleg with 10 µL OD_600_: 0.2 fresh bacteria in PBS and then observed for five days, with non‐inoculation and PBS injection as negative control. *moaD* (DK) and *moaE* (EK) knockouts showed less death than *A. baumannii* ATCC 19606 (WT), *moaD* (DC), and *moaE* (EC) complemented strains. *A. baumannii* ATCC 19606 (WT) and *moaE* (EC) complemented strain displayed highest lethality during five days, while MoaE C‐terminal truncation (ECtrunc) showed lethality intermediate between EK and EC, with no more death after the first two days of infection (Figure 5A,B).

**FIGURE 5:**
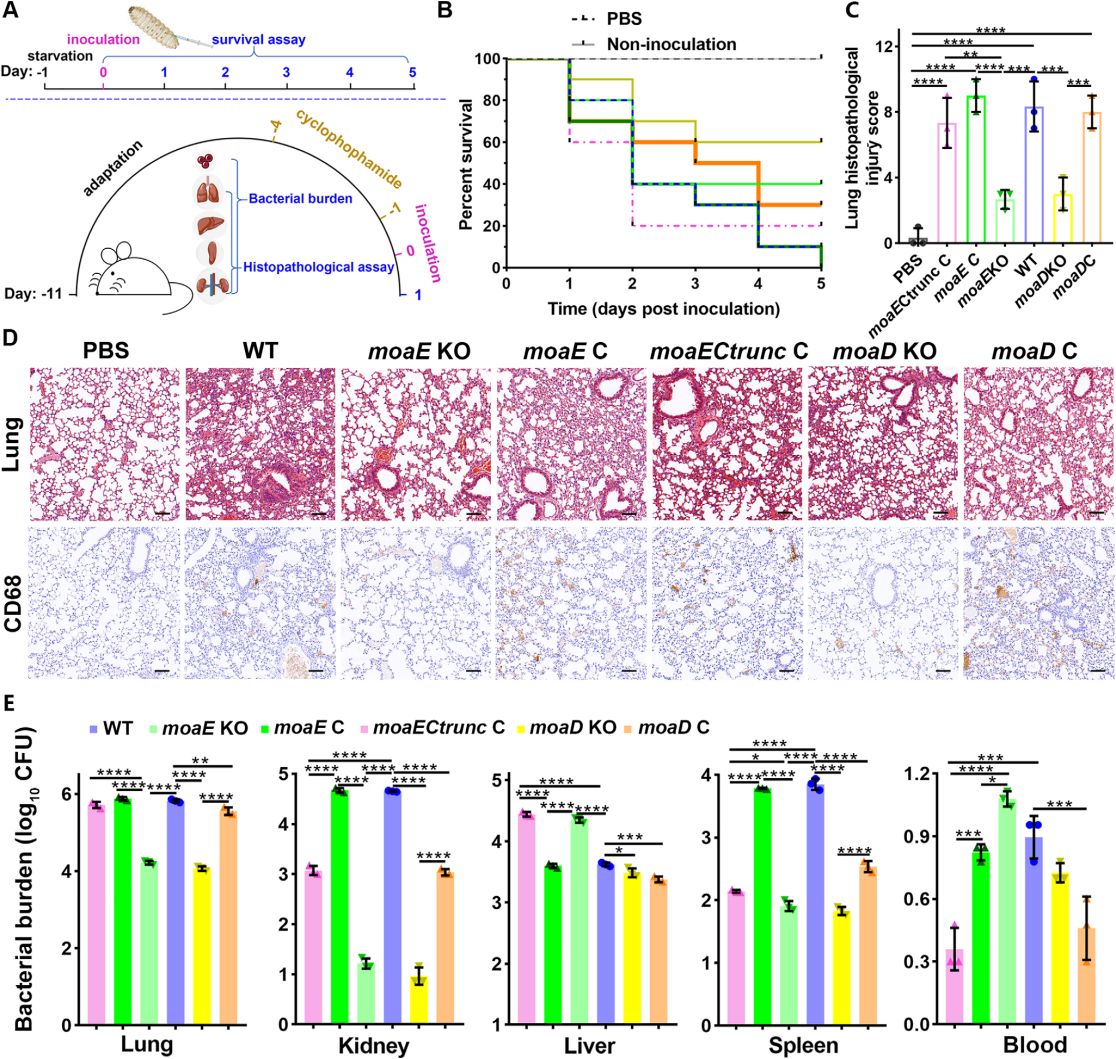
MPT synthase is required for A. baumannii full virulence in the infection of Galleria mellonella or mouse. (A) Sketch map of sample collection at 1 day after infection in mice nasal feeding infection model, blood, lung, spleen, liver and kidney were collected for bacterial burden and lung, spleen, liver and kidney were collected for histopathological analysis. (B) Survive curve of *Galleria mellonella* infection model. (C) Histopathological injury score of lungs by blinded evaluation. Statistics significance was calculated by one‐way ANOVA on GraphPad Prism. *p* < 0.01 was indicated by double asterisks, *p* < 0.001 was indicated by three asterisks, *p* < 0.0001 was indicated by four asterisks. (D) Histopathological analysis of the lung tissues from WT, EK, EC, ECtrunc, DK, DC and PBS 1 day after inoculation. *A. baumannii* infection caused inflammation infiltrates in the alveoli lumen, larger area of inflammation was observed in WT, EC and DC compared to EK and DK, respectively. The area of inflammation and inflammation cell counts of ECtrunc displayed larger and more than EK strain, while smaller and fewer compared to WT or EC. The macrophages recruitment was induced by bacterial invasion indicated by CD68 as brown. *Bars* = 100 µm. (E) Bacterial load of WT, EK, EC, ECtrunc, DK and DC strains (each group with 3 mice, respectively) in the lungs, blood, spleens, liver and kidney of the mice 1 day post intranasal inoculation. In lung, EK (4.22 ± 0.05) and DK (4.06 ± 0.06) significantly reduced bacterial load compared to WT (5.82 ± 0.04) or complemented strains EC (5.87 ± 0.04), ECtrunc (5.71 ± 0.08) or DC (5.55 ± 0.10), with *p* < 0.0001 labeled by four asterisks for EK against ECtrunc and DK versus DC. The similar tendency of bacterial burden was observed in kidney and spleen compared to lung. While, bacterial load of EK is relatively higher in Liver (4.35 ± 0.04) and Blood (1.08 ± 0.04). Values represent means ± standard errors and all statistics significance was calculated by one‐way ANOVA on GraphPad Prism. *p* < 0.05 was indicated by one asterisk, *P* < 0.01 was indicated by double asterisks, *p* < 0.001 was indicated by three asterisks, *p* < 0.0001 was indicated by four asterisks.

One day after nasal inoculation of mice with 50 µL of approximately 5×10^7^ CFU fresh bacteria, lung, kidney, liver, spleen and blood were harvested for bacterial burden counting, and lung, kidney, liver, and spleen were also collected for histopathology analysis (Figure 5A).

In lung tissue, *A. baumannii* infection caused inflammatory infiltrate, indicative of severe pulmonary inflammation with increased numbers of inflammatory cell and substantial swelling of the interstitial spaces as detected in *A. baumannii* ATCC 19606 (WT), and *moaD* (DC) or *moaE* (EC) complemented strains, compared to *moaD* (DK) or *moaE* (EK) deficiency strains (Figure 5C,D). The extent of inflammation caused by carboxyl terminal truncated MoaE (ECtrunc) complemented strain was intermediate between that of *A. baumannii* ATCC 19606 (WT) or *moaE* (EC) complemented and *moaE* (EK) deficiency strains (Figure 5C,D). The consistent phenomenon was detected by macrophage marked by CD68 antibody (Figure 5D). There was no difference observed in kidney, liver, and spleen between different strains’ invasion and PBS inoculation (Figure S6A).

Strains after 24 h culture in LB medium at 37°C shown minor difference (Figure S6B), while *moaD* (DK) or *moaE* (EK) knockout strains showed significantly lower bacterial burden than *A. baumannii* ATCC 19606 (WT), and *moaD* (DC) or *moaE* (EC) complemented strains in lung and kidney. *moaE* (EC) complemented strain and the wildtype strain showed similar bacterial loads, significantly the highest levels of bacterial load amongst all the strains in lung, kidney and spleen. Bacterial load upon complementation with the C‐terminal truncated MoaE (ECtrunc) was significantly less than *moaE* (EC) complemented strain in kidney, spleen and blood. In liver, the bacterial burden of *moaE* (EK) deficiency and MoaE carboxyl terminal truncation (ECtrunc) complemented strains were significantly higher than other strains (Figure 5E).

## 3 Discussion

To adapt to changes in metal availability, pathogenic microorganisms develop metalloregulatory systems to overcome host nutrient immunity including metal limitation and intoxication [65]. *Acinetobacter baumannii* possesses high nickel biosorption ability [66], and histidine‐rich regions like the carboxyl terminal tail of MoaE in *Acinetobacter*. *NikR* (13835), a nickel‐responsive regulatory [42] was significantly down regulated by *moaD* or *moaE* knock‐out. Somehow, nickel supplementation helps the *moaE* mutant to grow and to compensate for the interruption of Moco biosynthesis. The de‐repression of high‐affinity uptake systems is a physiological response to metal limitation [67], *modA* was up‐regulated upon Moco deficiency caused by *moaE* or *moaD* mutation. As well as sulfate transporters, which may function as a non‐specific, energy‐saving alternative molybdate acquisition system.

The presence of millimolar concentrations of sodium molybdate can functionally rescue a homologous fungal Mo‐insertase defect [68]. Moco biosynthesis has impacts on the metabolism of other metal ions besides molybdenum, verified by the metal content changes of different mutant strains of MPT synthase, as well as numbers of the metal related genes. In plants, during molybdenum incorporation, copper can bind to molybdopterin dithiolene sulphur [64], and zinc has been identified as a cofactor of CNX1E and is essential for its catalytic activity [69], whereas magnesium is required for the activity of CNX1G [70] (Figure 6). *moaE* knockout down‐regulated *betI*, which is a repressor that regulates *betIBA* operon responses to osmotic stress, and the interaction with salts can eliminate its repression [51]. *moaE* in *A. baumannii* may function as a nickel reservoir and metal ion sensor to increase resistance to metal intoxication. Though the limitations imposed by incomplete complementation, the truncation of C terminal histidine tail of MoaE increased the growth of bacterial stimulated by additional metal ions, probably by attenuating the compensatory stress mechanisms they induce. However, the truncation decreased intracellular content of molybdenum but mild pathogenicity in our infection models.

**FIGURE 6:**
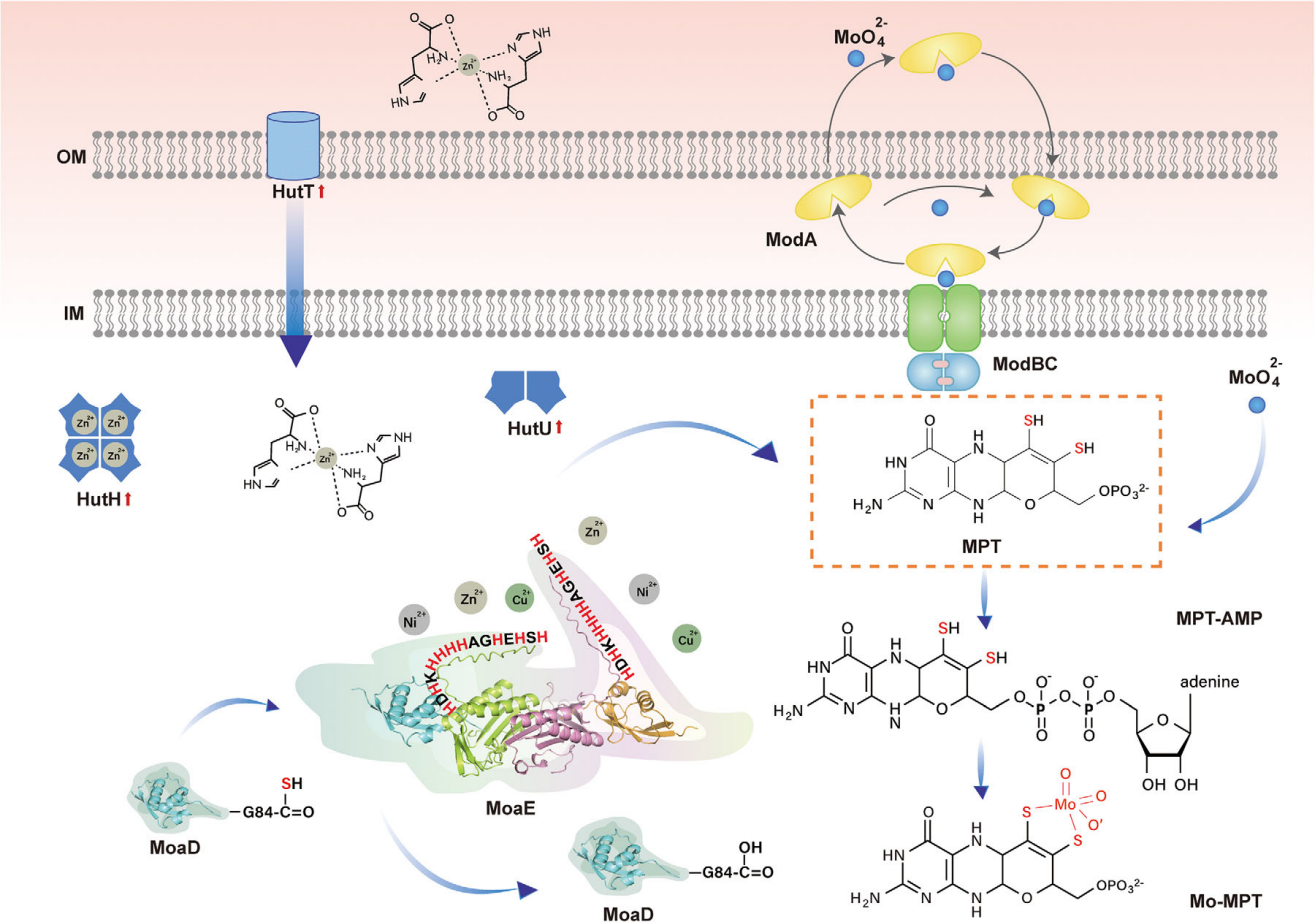
Schematic mechanism of MPT synthase involved in molybdate metabolism and metal homeostasis.

Iron is strongly linked to Moco biosynthesis in multiple ways: Fe‐S clusters are a component of many proteins related to Moco biosynthesis: MoaA; iron metabolism regulator FNR (fumarate and nitrate reduction), which regulates *moaABCDE* and *moeAB* operons, and IscS, which is required for sulfur transfer for many Fe‐S cluster‐containing proteins as well as Moco biosynthesis in MPT synthase. Many Moco‐containing enzymes also contain Fe‐S or heme cofactors [29, 71]. In our experiment, iron supplementation partially restored growth in *moaE* or *moaD* knockouts. We hypothesize that formation of Fe‐S clusters in many proteins was frustrated by non‐productive association of IscS with isolated MoaD or MoaE, and this could be partially alleviated by iron supplementation. It is also possible that certain Fe enzyme could potentially replace some functions of molybdenum enzymes to compensate for Moco biosynthesis deficiency. Further work is needed to clarify the exact mechanism of compensatory iron supplementation.

Lack of *moaE* increased *hut* operon expression, HutH binds Zn^2+^ tightly under high histidine concentration conditions, and can work as a labile His‐Zn pool [58], make it reasonable for light growth of *moaE* knockout strain under 600 µM zinc. Further, Mg^2+^ ions and the L‐histidine critically regulate *hutP*, the anti‐termination regulator of *hut* operon [72]. Mg^2+^ is required for re‐sulphuration of MoaD by MoeB [23], and insertion of molybdate into pterin‐based molybdenum cofactors [73]. The attenuation of growth of MoaE knockout is less severe than MoaD, and addition of zinc, magnesium and nickel recovered some extent growth of defective *moaE* in *A. baumannii*, which may be caused by indirect effects on metabolism or stress responses, contributing to metal dependent regulating of MoaE (Figure 6).

Knockout of *moaD* or *moaE* caused a significantly increased sensitivity to ROS, consistent with downregulation of *katE* [48], which are responsible for detoxifying hydrogen peroxide. The truncation of the histidine‐rich tail of MoaE increased resistance to hydrogen peroxide compared to intact MoaE. Metal ions may also be in charge, Cu^2+^/Ni^2+^‐binding motif, termed the ATCUN motif, which consists of a histidine residue at position 3 (XXH, where X represents any residue), while the Zn^2+^‐binding motif is HEXXH (where X represents any residue). The ATCUN motif adopts a square planar geometry, and upon metal ion binding, it has been shown to induce the production of reactive oxygen species (ROS) in the presence of ascorbic acid or magnesium monoperoxyphthalate [74, 75] (Figure 6). Further work is needed to determine whether metal ions coordinated by the MoaE His‐rich tag is able to catalyze ROS production.

Carboxyl terminal of MoaE may confer an optimal structural configuration to enable peptides to interact with other molecules. In *M. tuberculosis*, the plastic C‐terminal region of MoaE changes its interaction with MoaD partner, structural rearrangements and further the function of MPT synthase [26]. The histidine‐rich carboxyl terminus in UreE of *Klebsiella aerogenes* is responsible for nickel‐binding but not essential to urease activity [76]. Intriguingly, the binding affinity of MoaD to MoaE (939 nm) in inactive MPT synthase of *A. baumannii* is at similar level of MoaD‐SH to MoaE (2.6 ± 0.9 µM) in active state of *E. coli* [62]. As binding affinity of the two subunits has been found to correlate with MPT activity, MPT synthase in *A. baumannii* is probably more active. More works are needed to explore the exact function of the intrinsically disordered histidine‐rich carboxyl terminal tail of MoaE.

## 4 Material and methods

### 4.1 Sequence Similarity Networks Assay

PSI‐BLAST searches were conducted using the sequence of MoaD or MoaE as a query against the National Center for Biotechnology Information (NCBI) non‐redundant protein sequence (nr) and metagenomic protein (env_nr) databases until convergence [77]. 713 and 1400 protein sequences were retrieved for MoaD and MoaE, respectively. Then homologs belong to *Acinetobacter*, with 686 for MoaD or 1000 for MoaE, were manually curated.

The Enzyme Function Initiative‐Enzyme Similarity Tool was performed to calculate the similarity between all protein sequence pairs [78], with identity at least 40%. 28 or 401 nodes and 318 or 79798 edges with 100% edge percentage identity sequence similarity networks of MoaD or MoaE was constructed, then the organic layout of Cytoscape (v3.9.1) was explored and visualized [79].

### 4.2 MoaE C‐Terminal Histidine Evolutionary Analysis

Homologs were identified with one sequence in one species of bacteria by sequence similarity and confirmed by PF02391/IPR003448 domain annotations [80], with MoaE (WP_000657202.1) from *Acinetobacter baumannii* ATCC 19606 as the reference. Native C‐terminal extensions were retained, records annotated as partial or fragmentary, sequences lacking a complete MoaE core, obvious truncations over 20%, the percentage of X‐rich over 1% or X is more than three, supported artificial tags, and proteins assigned to MoeA, MoaD, MogA, ModA, MoaB, MoaC, MobA, MobB, ThiS and ThiF were excluded. Taxonomy was resolved against NCBI Taxonomy [81], and one sequence was retained per species using a fixed hierarchy based on reference status, genome completeness, annotation quality, core coverage and accession order.

For each selected proteins, the final 18 residues of the original unaligned sequence were extracted directly, domain boundaries were not used. We calculated H_count, and residue frequencies at positions −18 to −1. Genus‐level summaries were restricted to genera represented by at least 30 species and included 10,000‐replicate percentile‐bootstrap intervals for mean H_count. *Acinetobacter* was compared with the remaining bacterial species representatives using the Mann–Whitney U test [82] and Cliff's δ [83]. Frequency logos were based on amino‐acid frequency using Shannon entropy and a finite‐sample correction [84].

### 4.3 Construction of A. Baumannii Gene Knock‐Out and Complemented Strains

*moaD* or *moaE* knock‐out strain was constructed as previously described[8]. 500 bp upstream and downstream of *moaD* or *moaE* were amplified from *A baumannii* ATCC 19606 genomic DNA, connected with kanamycin cassette from pKD4 plasmid through homologous fragment recombination via seamless cloning into pET28a vector. Confirmed by DNA sequencing, PCR products of *moaD* or *moaE* “upstream‐kanamycin cassette‐downstream” fragments were transformed into *A. baumannii* ATCC19606 with pAT04 expressing REC_AB_ to replace the original *moaD* or *moaE* locus by electroporation method. ID primers were used to harvest the sequence and validate the knock‐out strains. Complemented strains were constructed by pAT04 vector recombination with *moaD*, *moaE* or C‐terminal truncated MoaE (ECtrunc) induced by IPTG, with *ECtrunc* generated by mutant D143(GAT) to termination codon (TGA) of *moaE*. All the primers used are listed in Table S2.

### 4.4 Bacterial Observation via Scanning Electron Microscopy

Each strain was cultured with shaking at 37°C in LB overnight, then diluted to OD_600_ 0.1, transferred into a 12‐well plate, with 1 mL in each chamber soaking a sterile slide, then cultured still for 24 h at 37°C. The bacteria on the slides were first fixed by 4% paraformaldehyde for 20 min at room temperature, and further fixed by 2.5% glutaraldehyde overnight at 4°C. Phosphate buffer was used to wash the slides four times, with 15 min for each time. 30%, 50%, 70%, 80% to 90% increasing gradient ethanol were performed for dehydration, with each step for 15 min. At last slides were dehydrated in 100% ethanol for three times and each for 30 min. The samples were observed under MAIA3LMH (TESCAN, Czech Republic) after sputter‐coating with gold. Widths were calculated from bacterial images using Image J, and statistical analysis was performed in GraphPad using three independent biological replicates.

### 4.5 Cloning, Expression, and Purification of Recombinant MoaD, MoaE and the C‐Terminally Truncated MoaE (ECtrunc)

For protein purification, a recombinant vector of pET28a‐*Ab*MoaD was built by homologous recombination using 2x MultiF Seamless Assembly Mix (ABclonal) after amplification from *A baumannii* ATCC 19606 genomic DNA. pET28a‐MoaE was built by double digestion with NcoI and EcoRI restriction enzymes after *moaE* fragment amplification. The C‐terminal truncated mutant MoaE (ECtrunc) was further generated by mutant D143(GAT) to termination codon (TGA) of *moaE*. All primers used are listed in Table S2. Recombinant protein expression in *E. coli* strain BL21(DE3) was induced by 0.5 mM isopropyl β‐D‐1‐thiogalactopyranoside (IPTG) at OD_600_ 0.6∼0.8 at 37°C, meanwhile transferred to 18°C culturing for around 16 h. The cell pellets were collected at 4°C by centrifugation at 6000×g for 10 min, then resuspended by Buffer A (20 mM Tris, 150 mM NaCl, 5% glycerol, pH 8.0), followed by ultrasonication. After removing cell debris, the lysate was loaded to an Ni‐NTA agarose column (Qiagen) and equilibrated with Buffer A, washed with Buffer A containing 25 mM imidazole, then eluted by Buffer B (20 mM Tris, 150 mM NaCl, 5% glycerol, 500 mM imidazole, pH 8.0). A Superdex 75pg, Hiload 16/60 (GE Healthcare Life Sciences) size exclusion chromatography column was used for further purification, equilibrated with Buffer A. Protein purification was conducted at 4°C.

### 4.6 H_2_O_2_ Tolerance Assay

H_2_O_2_ resistance was performed by disc diffusion assay [59], the bacteria were cultured in LB overnight at 37°C, 220 rpm, then diluted to OD_600_: 0.2, 200 µL of which were mixed LB‐agar that was melted and cooled before solidification, with 2 mM IPTG and appropriate antibiotic added. Three petri dishes were made using 50 mL LB‐agar, then a fixed size sterile circle filter paper having 10 µL 0.22 M H_2_O_2_ was placed on the center. The inhibition zone was measured by the diameter after overnight culture in 37°C. GraphPad Prism was performed to analyze the data from three biological repeats.

### 4.7 Biofilm Formation Assay

Biofilm biomass was detected by crystal violet staining with modifications [85]. Bacteria were cultured in LB broth overnight at 37°C, 220 rpm, then diluted to OD_600_ = 0.1, 200 µL of which were then inoculated into 96 well plates with 2 mM IPTG and appropriate antibiotic, and cultured for an additional 24 h at 37°C. 150 µL bacteria were transferred into new 96 well plate and measured at OD_600_. The plate was gently washed by PBS for three times, then stained by 1% crystal violet for 15 min. The plate was washed by PBS for three times, and then solubilized with 95% ethanol and measured at OD_570_. Three wells were inoculated for each strain and three independent biological repeats were conducted.

### 4.8 The Influence of Metal Ions on Bacterial Growth

1 L LB‐agar medium liquid was prepared and homogeneous mixing, then divided into 100 mL per conical flask, 80 µM, 180 µM, or 600 µM metal ions in each conical flask, was diluted from 1 m stock solution of different metal ions, without significant change in pH. After autoclaved sterilization, the medium was poured into petri dish in addition of 2 mM IPTG and appropriate antibiotic. Overnight cultured bacteria in LB broth were diluted to OD_600_ = 0.1, then 10 µL of which were point inoculated on the plate. Bacterial counting was demonstrated after overnight culture at 37°C. Three independent biological repeats were conducted and, collected data was analyzed on GraphPad Prism.

### 4.9 RNA Sequencing and Analysis

Bacteria of WT, DK, DC, EK, EC or ECtrunc strains were cultured to OD_600_ = 0.6 from a single colony. After collection of bacteria, total RNA extraction was performed by TRIzol Reagent/RNeasy Mini Kit (Qiagen), then the quantity and quality of the total RNA was assessed with an Agilent 2100/2200 bioanalyzer (Agilent Technologies, Palo Alto, CA, USA), NanoDrop (Thermo Fisher Scientific Inc.) and 1% agarose gel. After the treatment of rRNA Removal Kit according to the manufacturer's instructions, 1 µg total RNA was used to construct a next generation cDNA library. The high sequencing depth of more than 14.5 million reads per sample was generated when libraries were loaded on an Illumina HiSeq. Cutadapt (v1.9.1) was performed to filtered the raw reads to abandon the adaptors and low‐quality bases [86], then were mapped to *A. baumannii* strain ATCC 19606 chromosome complete genome (accession CP046654) by bowtie2 (v2.2.6).

Significant differential expression genes were calculated through DEGSeq (v1.38.0) [87], based on two criteria: ∣log2 fold change∣> 1 and adjusted *p*‐value (*p*‐adj) < 0.05. Gene expression pattern heat map was clustered through hierarchical clustering according to Z‐score normalized gene count. KEGG pathway assay was displayed with the number of DEGs ≥ 2 distributed by the rich factor.

### 4.10 Quantitative RT‐PCR

The bacteria of WT, EK, EC, ECtrunc, DK and DC cultured to OD_600_ = 0.6 from a single colony was harvested. Total RNA was isolated using RNAprep Pure Total RNA kit for Cultured Cells/Bacteria (spin‐column‐based, Cat. No. DP430, TIANGEN, Beijing, China), during procedure genomic DNA was removed by DNase I digestion. RNA concentration and purity were determined by NanoDrop spectrophotometer, and RNA integrity was verified by agarose gel electrophoresis. First‐strand cDNA was synthesized from 1 µg of total RNA by HiScript IV All‐in‐One Ultra RT SuperMix for qPCR (Cat. No. R433‐01 Vazyme, Nanjing, China).

qRT‐PCR was performed using SupRealQ Ultra Hunter SYBP qPCR Master Mix (U+) (Cat. No. Q713‐02, Vazyme, Nanjing, China) in a total reaction volume of 20 µL. Thermal cycling was conducted on a Bio‐Rad CFX96 C1000 Touch Thermal Cycler with the conditions: initial denaturation at 95°C for 3 min; 40 cycles of 95°C for 10 s, 57°C for 30 s. Melting‐curve analysis (65°C to 95°C, 0.5°C increment per step) was performed after amplification to confirm primer specificity. No‐template control (NTC) was included in each run.

The housekeeping gene 16srRNA was used for normalization [88]. Three independent biological replicates were performed with three technical replicates. Relative gene expression was calculated by the 2^‐ΔΔCt method. Statistical analyses were carried out using GraphPad Prism based on ΔCt. Primers are listed in supplementary Table S2.

### 4.11 ICP‐MS for Intracellular Metal Levels

Strains were harvested at OD600 = 0.6 for metal ions measurement, inactivated by autoclave, the weight was recorded after 60°C drying and filtration. After digesting by nitric acid in PTFE tube using Touchwin2.0, dilution by ultrapure water in PET plastic bottle, metal levels were detected through ICP‐MS (PE NexION 300D) [89].

### 4.12 Isothermal Titration Calorimetry (ITC) Analysis of MoaD Binding to MoaE

Purified recombinant MoaD and MoaE were diluted to 200 µM and 20 µM, respectively. The stock solution Buffer A was from the size exclusion chromatography during MoaD and MoaE proteins purification. MicroCal PEAQ‐ITC (Malvern) was used to perform ITC assay at 25°C following instructions [8]. 100 µL MoaD was titrated into 300 µL MoaE in the sample chamber. MicroCal PEAQ‐ITC analysis software (Malvern) was used for data analysis after three independent biological replicates. For metal ions interaction assay all proteins were expressed with or without His‐tag and chelated by 1 mm EDTA prior to purification by size exclusion chromatography, MoaE was purified through Ni‐NTA via its histidine‐rich carboxyl terminus, while MoaE Ctrunc was purified through ion exchange chromatography. Metal ions were prepared at 200 µm and proteins were diluted to 20 µM.

### 4.13 Thermal Shift Assay of MoaE or MPT Synthase in Response to Metal Ions

20 µm purified recombinant MoaE, MPT synthase (MoaDE_2_) or MoaE with carboxyl terminal truncation protein was incubated at 4°C for about 30 min with ZnCl_2_, NiSO_4_ or CuCl_2_ at concentration of 5, 20 or 80 µm, respectively. Then mixed with the dye from a protein thermal shift dye kit (Thermo Fisher Scientific Applied Biosystems), the solution was run on a Bio‐Rad CFX96 C1000 Touch Thermal Cycler to detect fluorescence intensity during an increase of temperature from 25°C to 98°C with 0.1°C step size. Three independent biological replicates were conducted and data was analyzed on GraphPad Prism (La Jolla, CA, USA).

### 4.14 Crystallization, Data Collection and Processing, and Structure Determination

Sitting drop vapor diffusion method were conducted for initial crystallization screening experiments through 384 well plates of commercially available sparse matrix screen kits (Molecular Dimensions and Hampton Research) at 20°C. 0.5 µL protein solution mixed with the same volume of reservoir solution equilibrating against 45 µL reservoir solution. After purification of MoaD and MoaE, MoaE was mixed with excessive mole number of MoaD. The concentration of MPT synthase was 40 mg/mL for crystallization, crystals were found in CSI+II G8, with 0.1 m Sodium chloride, 0.1 m HEPES pH 7.5, and 1.6 m Ammonium sulfate.

Datasets were collected at Shanghai Synchrotron Radiation Facility Beamline 18U1 and 19U1, then XDS were performed to process and scale the X‐ray diffraction data [90]. Data collection details and statistics are summarized in Table S3. Maximum‐likelihood molecular replacement was implemented through the Phaser program suite to determine the structure of MPT synthase with 6jbz [91] as search model with MoaD or MoaE sharing 22% or 35% identity, respectively. Manual model rebuilding and refinement were performed via PHENIX and COOT [92, 93]. At last, the crystal structure of MPT synthase was solved at 1.97 Å in spacegroup P4_1_2_1_2 with cell dimensions a = 109.4 Å, b = 109.4 Å, c = 121.4 Å and α = 90.0 °, β = 90.0 °, γ = 90.0 °, the structure was refined to a final Rwork/Rfree of 0.1930/0.2210. Pymol program version 2.5 by Schrödinger was used to generated all structure illustrations. The protein interfaces were generated through PDBePISA https://www.ebi.ac.uk/pdbe/pisa/.

### 4.15 Small Angle X‐Ray Scattering (SAXS)

MPT synthase was purified fresh from size exclusion chromatography with the concentration of 5 mg/mL. SAXS data was collected under batch mode at 293 K, with a moment transfer range from 0.04 to 0.45 Å^−1^, at the Shanghai Synchrotron Radiation Facility BL19U2 Beamline, summarized in Table S7. Twenty frames were collected without detectable radiation damage for each sample. Data interpretation was conducted through ATSAS 3.0, buffer subtraction was performed by PRIMUS, then harvested P(r) by Distance Distribution assay, or molecular weight determination by Porod Volume or Molecular Weight assay [94] as well as MoW2 server [95]. The comparison of the crystal structure to SAXS experimental data was conducted on DAMMIF, and CORAL on web services by adding missing carboxyl histidine‐rich terminal of MoaE.

### 4.16 Galleria Mellonella Infection Model of A. Baumannii

The *Galleria mellonella* infection experiment was conducted following previous reports [96], the larval stage of *Galleria mellonella* with length of 2–3 cm, weight of 250–350 mg were kept for experiment, 24 h starvation was performed before injection. After overnight culture, the bacteria were diluted to OD_600_ = 0.2 by sterile PBS, 10 µL of which were injected into the larval body at last left proleg, with non‐inoculation and PBS injection as negative control. Then, ten larval of each group were kept in one square petri dish at 37°C with the same regular feeding, and were checked for activity every day till five days post injection. Survival curves were generated using GraphPad Prism.

### 4.17 Baumannii Murine Model of Acute Pneumonia

Six‐week‐old C57BL/6JNifdc male mice were raised in the same situation for one week adaptation to further experiment. To induce neutrophil deficiency, 150 mg/kg cyclophosphamide was used twice to pretreat mice at day 4 and day 1 before bacterial inoculation [8]. *A. baumannii* strains were prepared by three times washing with saline, and then diluted to appropriate optical density after culture at 37°C overnight, different groups were weight matched, then each mouse under anesthesia by isoflurane was intranasally inoculated with about 5×10^7^ CFU bacteria in 50 µL saline. Three infected mice from each group were sacrificed humanely at one day after inoculation, blood, lung, spleen, liver and kidney were harvested for further quantitative bacteriology or histopathology assay.

Blood samples as well as homogeneity of lung, spleen, liver and kidney with 500 µL sterile saline through homogenizers (MiNiBeadbeater‐16, BioSpec Products Inc. USA), were 10‐fold diluted, and 10 µL from which was inoculated on LB agar plates cultured overnight for bacterial counting. Samples of lung, spleen, liver and kidney were fixed in 4% paraformaldehyde upon collected immediately, then demonstrated standard paraffin embedding methods to prepare the paraffin blocks. The tissue slices stained by hematoxylin and eosin (HE) were observed by fully automatic digital slicing scanner (Pannoramic DESK, 3DHISTECH, Hungary) for histopathology assay after slice.

Histopathological injury score of lungs in a blinded manner as reported [97]. Briefly, 8–10 random high‐power fields per mice were evaluated. Five parameters including interstitial inflammatory infiltration, alveolar exudation, pulmonary edema, alveolar hemorrhage, and alveolar structural damage were each scored from 0 to 4 (0 = no injury; 1 = < 25% injury; 2 = 25–50% injury; 3 = 50–75% injury; 4 = > 75% injury). The sum of individual item scores represented the total histopathological score for each field, and the average value of all fields was used as the final score for each mouse.

## Author Contributions

YW and MJ designed and conducted the project, MJ, WH, ZO, RW, JZ, XL, YJ, QY, XH, and QQ carried out experiments under the supervision of YW. LG, FZ, PH involved in the data analysis. YW and MJ wrote the paper with contribution from all the authors.

## Conflicts of Interest

The authors declare no conflicts of interest.

## Ethics Statement

All applicable institutional and/or national guidelines for the care and use of animals were followed and all animal experiments were approved by the Ethical Committee for Animal Experiments of Xi'an Jiaotong University (NO. XJTUAE2025‐71).

## Supporting information




**Supporting File**: advs78547‐sup‐0001‐SuppMat.pdf.

## Data Availability

The data that support the findings of this study are available on request from the corresponding author. The data are not publicly available due to privacy or ethical restrictions. Accession number The structure factors and coordinates of MoaDE complexed with SO_4_^2−^ and Mg^2+^have been deposited in the Protein Data Bank under the accession code 9XGP.
