# Supplementary material for: The Interplay Between Molybdopterin Synthase and Metal Metabolism Contributes to Pathogenicity in Acinetobacter baumannii
Source: Adv Sci (Weinh). 2026 Oct 29:e78547. Online ahead of print. doi: 10.1002/advs.78547 (PMC13720259; doi:10.1002/advs.78547)
Supplement: Supplementary file 1 [file ADVS-9999-e78547-s001.pdf]

Figure S1

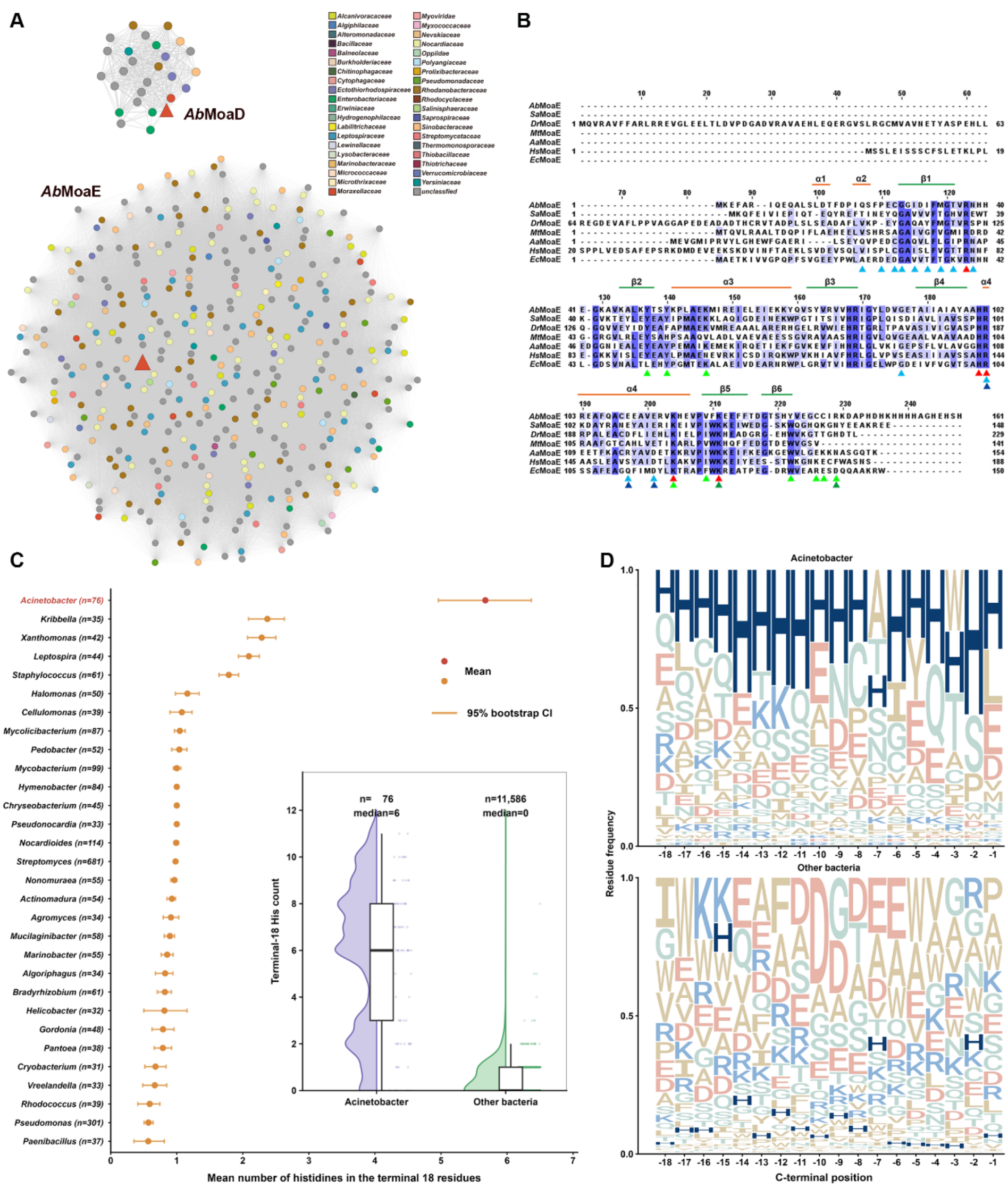

Fig. S1. Phylogenetic analysis of *Ab*MoaD and *Ab*MoaE.

(A) Sequence similarity network shown *Ab*MoaD at the edge, while *Ab*MoaE close to the center connected with 28 or 401 nodes and 318 or 79798 edges, respectively, with a threshold of PSI-Blast 40% sequence identity homologs excluding in *Acinetobacter* were chosen. Total 712 homologs were collected including 685 belonging to *Acinetobacter* to *Ab*MoaD, while 1399 homologs were generated with 999 from *Acinetobacter* to *Ab*MoaE.

(B) Sequences alignment of *Ab*MoaE with other top six structural similar MoaEs generated by Dali search, with secondary structure labeled above by orange and green for α helix and β sheet, respectively. Interface amino acids between *Ab*MoaE and *Ab*MoaD, *Ab*MoaE dimers were marked by triangle with green or dark green, blue or dark blue for hydrogen bonds or salt bridges. The predicted substrate binding amino acid was marked by red triangle. The amino acid of active site was labeled by red triangle based on MPT structure of *Staphylococcus aureus*.

(C) *Acinetobacter* shown marked C-terminal histidine enrichment. Among genera represented by at least 30 species of bacteria, *Acinetobacter* ranked first for mean histidine count shown by top 30. In the complete species-representative bacterial data set, the median H_count was 6 (IQR 3–8) in

Acinetobacter and 0 (IQR 0–1) among 11,586 other bacteria (Mann–Whitney U=876,024; Cliff's δ=0.862)

(D) C-terminal histidine of last 18 amino acids distribution of MoaE in *Acinetobacter* (top) or other bacteria (bottom) by frequency logos.

Figure S2

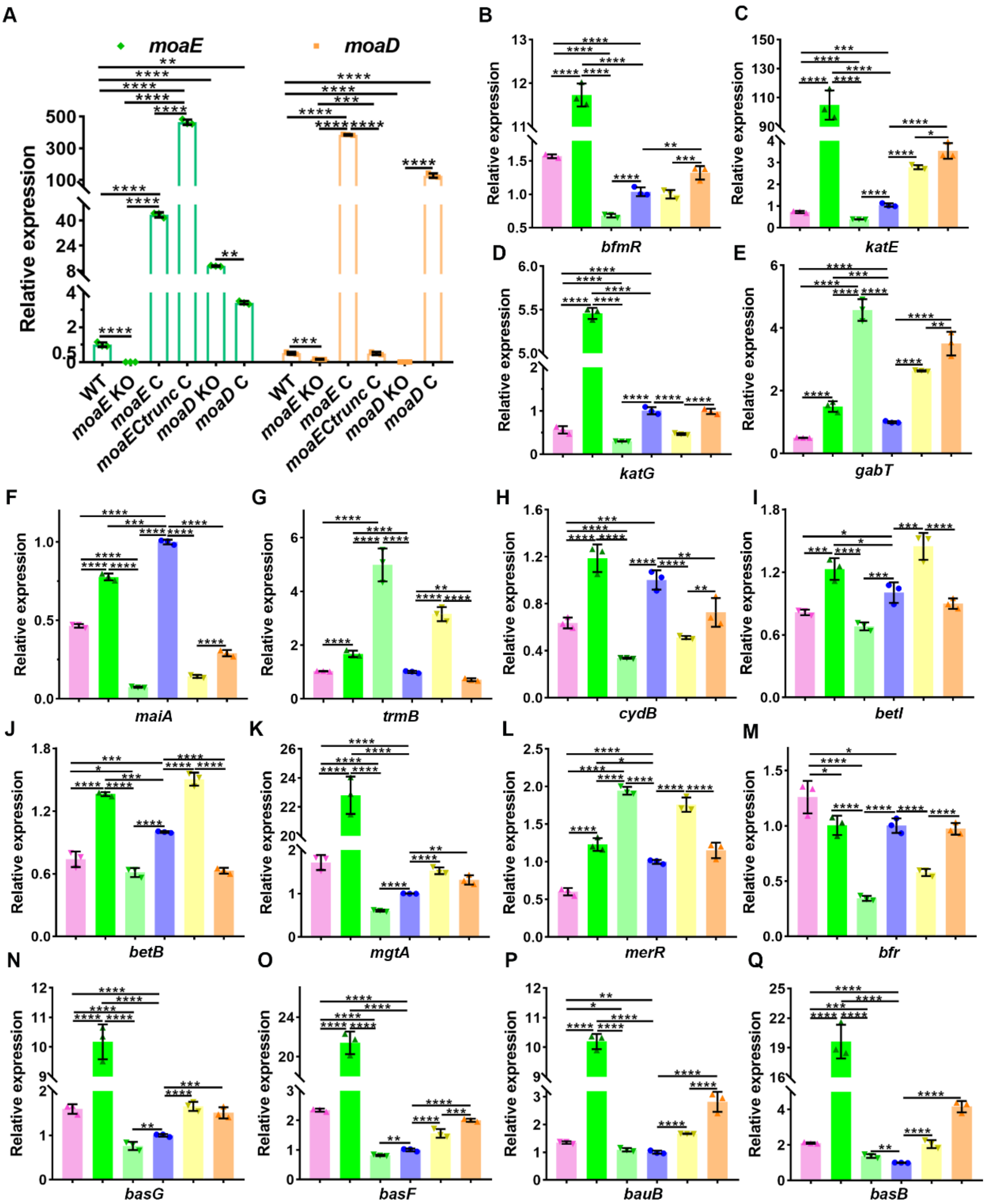

Fig. S2. The relative gene expression level validated by qRT-PCR.

All statistics significances were calculated based on ΔCt by one-way ANOVA via GraphPad Prism, with four asterisks indicating $P < 0.0001$, three asterisks for $P < 0.001$, two asterisks for $P < 0.01$, and one asterisk for $P < 0.05$.

Figure S3

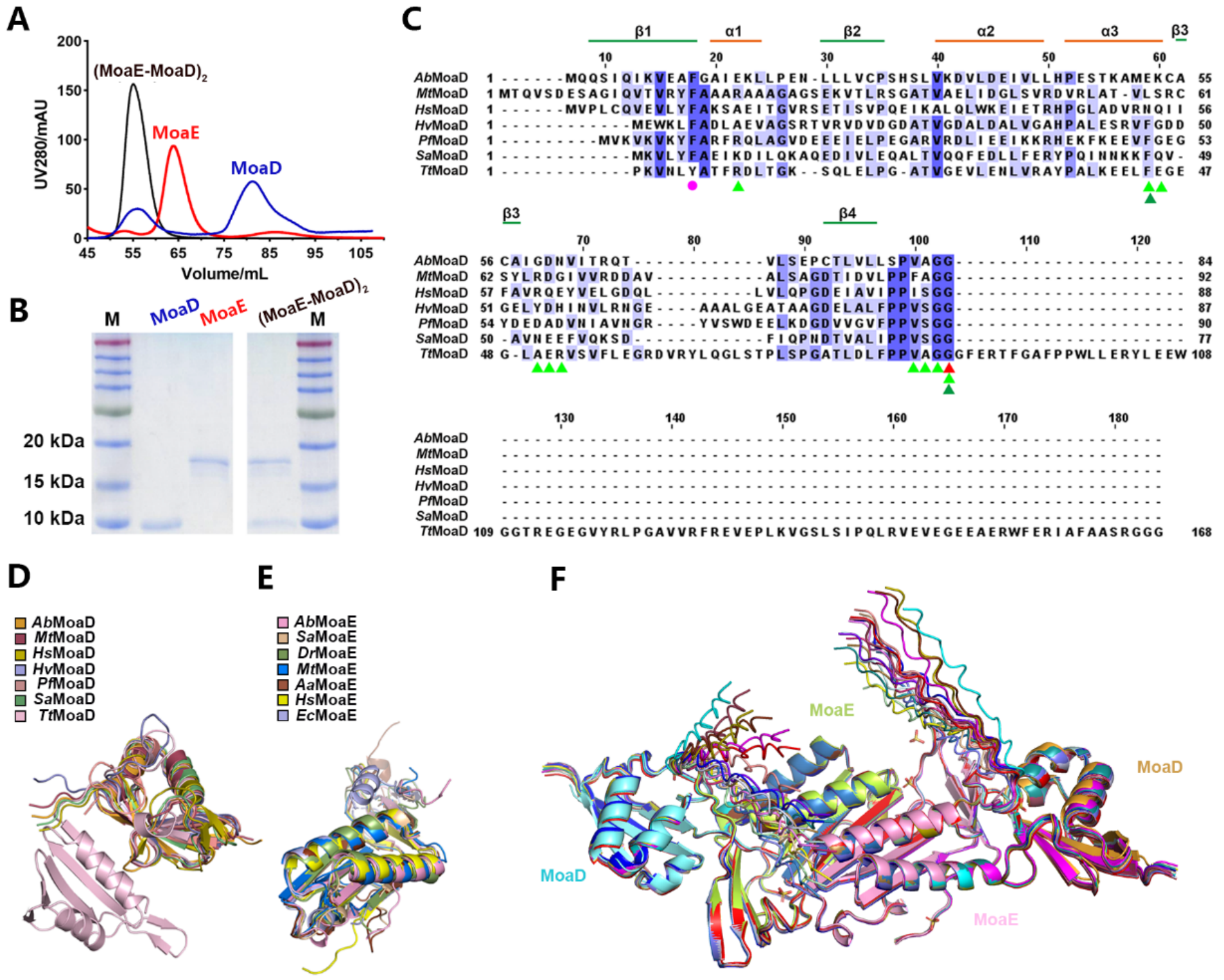

Fig. S3. The purification, sequential and structural characteristics of MPT synthase.

(A) *Ab*MoaD, *Ab*MoaE and MPT synthase were purified by size exclusion chromatography column HiLoad TM 16/600 superdex 75pg, the peak at 81.06 mL, 61.32 mL and 57.25 mL, repectively indicates a monomer, dimer and hetero-tetramer solute protein, combined with the SDS-PAGE results.(B) The purified *Ab*MoaD, *Ab*MoaE and MPT synthase proteins were confirmed the size of 10 kDa,18 kDa and mixture of 10 kDa and18 kDa through SDS-PAGE by coomassie blue staining.(C) Sequences alignment of *Ab*MoaD with other top six structural similar MoaDs generated by Dali search, with secondary structure labeled above by orange and green for α helix and β sheet, respectively. Interface amino acids between *Ab*MoaD and *Ab*MoaE were marked by triangle with green or dark green for hydrogen bonds or salt bridges. The predicted substrate binding amino acid was marked by red triangle. The amino acid in hydrophobic was labeled by pink circle based on MPT structure of *Staphylococcus aureus*. (D) Superposition of *Ab*MoaD with its top six structural similar MoaDs generated by Dali search, with RMSD of 1.148 (395 atoms), 1.654 (422 atoms), 1.41 (382 atoms), 1.465 (416 atoms), 1.441 (405 atoms) and 1.408 (341 atoms) for *Mt*MoaD, *Hs*MoaD, *Hv*MoaD, *Pf*MoaD, *Sa*MoaD and *Tt*MoaD against *Ab*MoaD, respectively.

(E) Structure alignment of *Ab*MoaE with its top six structural similar MoaEs generated by Dali search, with RMSD of for 0.757 (89 atoms), 1.069 (106 atoms), 1.053 (114 atoms), 0.638 (92 atoms), 1.069 (111 atoms), 0.974 (90 atoms) for *Sa*MoaE, *Dr*MoaE, *Mt*MoaE, *Aa*MoaE, *Hs*MoaE and *Ec*MoaE against *Ab*MoaE, respectively.

(F) Structure alignment of MPT synthase based on crystal with each subunit labeled consistently with Fig 3B and 20 structures predicted from AlphaFold 3 marked by red, raspberry, salmon, warmpink, chocolate, blue, slate, skyblue, purpleblue, wheat, palegreen, bluewhite, deepteal, cyan, palecyan, yellow, sand, magenta, violet and olive.

Figure S4

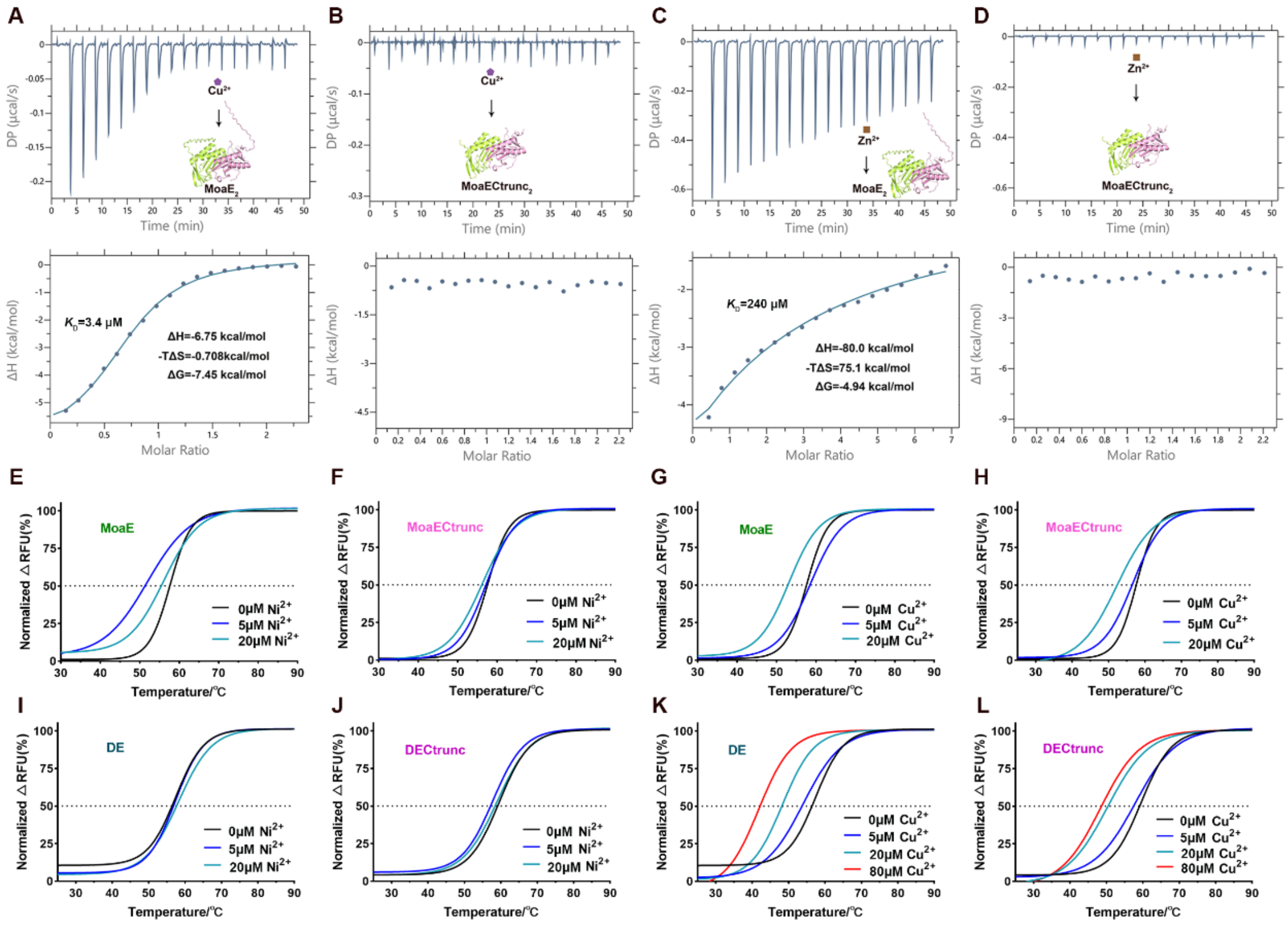

Fig. S4. The carboxyl histidine-rich terminal of MoaE involves in interaction with Cu^{2+} or Zn^{2+}.

(A) Isothermal titration calorimetry experiment indicates the thermodynamics of the interaction of *Ab*MoaE with Cu^{2+}. (B) The truncation of histidine-rich carboxyl terminal of *Ab*MoaE abolish its interaction with Cu^{2+} detected by isothermal titration calorimetry. (C) Isothermal titration calorimetry experiment indicates the thermodynamics of the interaction of *Ab*MoaE with Zn^{2+}.

(D) The truncation of histidine-rich carboxyl terminal of *Ab*MoaE abolish its interaction with Zn^{2+} detected by isothermal titration calorimetry. (E) Heat shift assay shows 20 μM or 5 μM Ni^{2+} decreases the T_m of MoaE dimer, the decrease extent of 5 μM is stronger than 20 μM.

(F) Serials of Ni^{2+} shows no significant change of T_m of MoaE dimer after histidine-rich carboxyl terminal truncation by heat shift assay. (G) Heat shift assay shows 20 μM Cu^{2+} decreases the T_m of MoaE dimer. (H) Heat shift assay shows 20 μM Cu^{2+} decreases the T_m of MoaE dimer with histidine-rich carboxyl terminal truncated. (I) Serials of Ni^{2+} shows no significant change of T_m of MPT synthase by heat shift assay. (J) Serials of Ni^{2+} shows no significant change of T_m of MPT synthase after histidine-rich carboxyl terminal truncation by heat shift assay. (K) Heat shift assay shows serials of Cu^{2+} decreases the T_m of MPT synthase, the decrease extent grows as Cu^{2+} density increases. (L) Heat shift assay shows serials of Cu^{2+} decreases the T_m of MPT synthase after histidine-rich carboxyl terminal truncation, the decrease extent of 80 μM is closed to 20 μM.

Figure S5

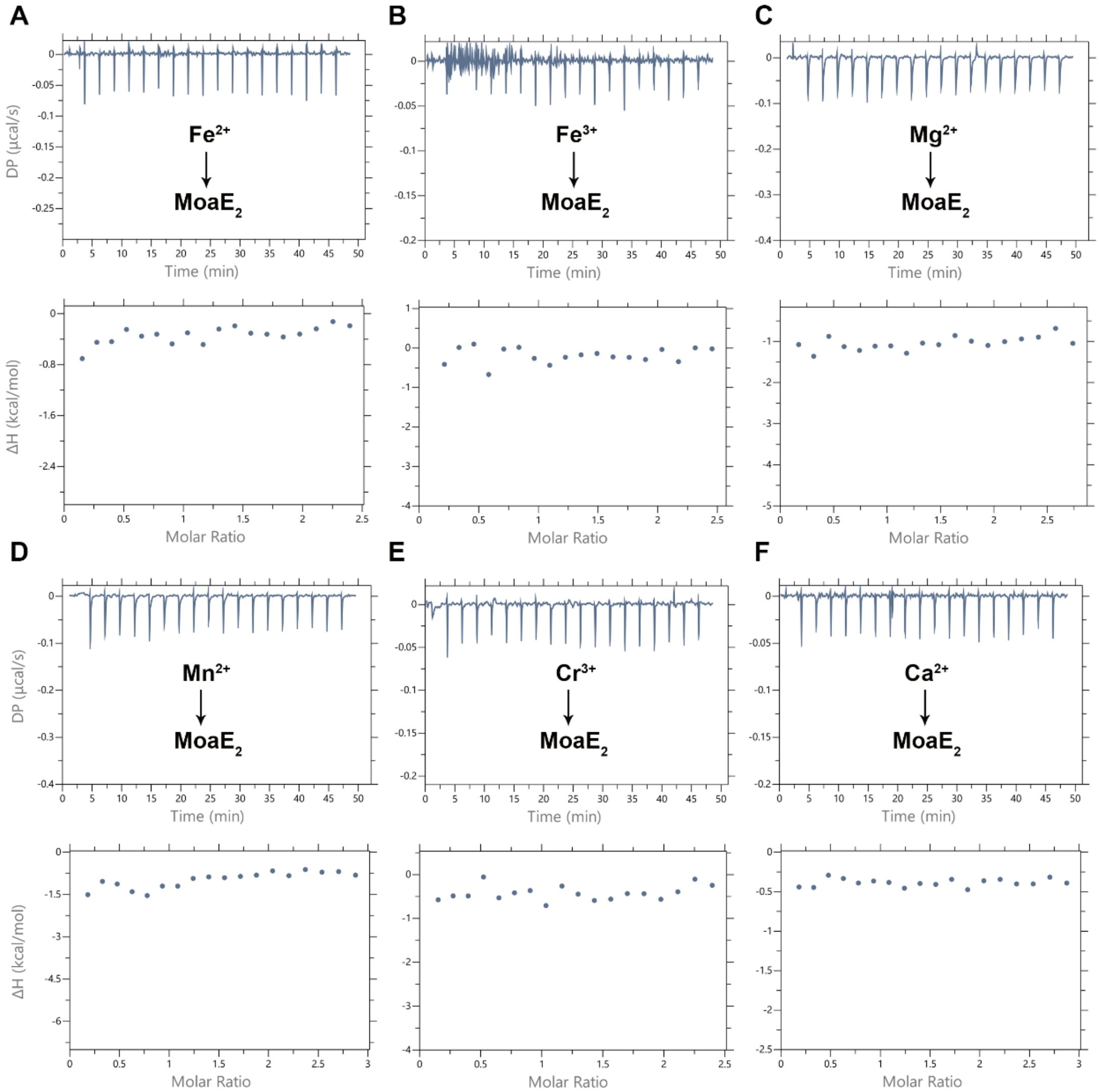

Fig. S5. No interaction was detected of MoaE with Fe^{2+}, Fe^{3+}, Mg^{2+}, Mn^{2+}, Cr^{3+} or Ca^{2+}.

Isothermal titration calorimetry experiment indicates no interaction of *Ab*MoaE with Fe^{2+}(A), Fe^{3+}(B), Mg^{2+} (C), Mn^{2+} (D), Cr^{3+}(E), and Ca^{2+}(F).

.

Figure S6

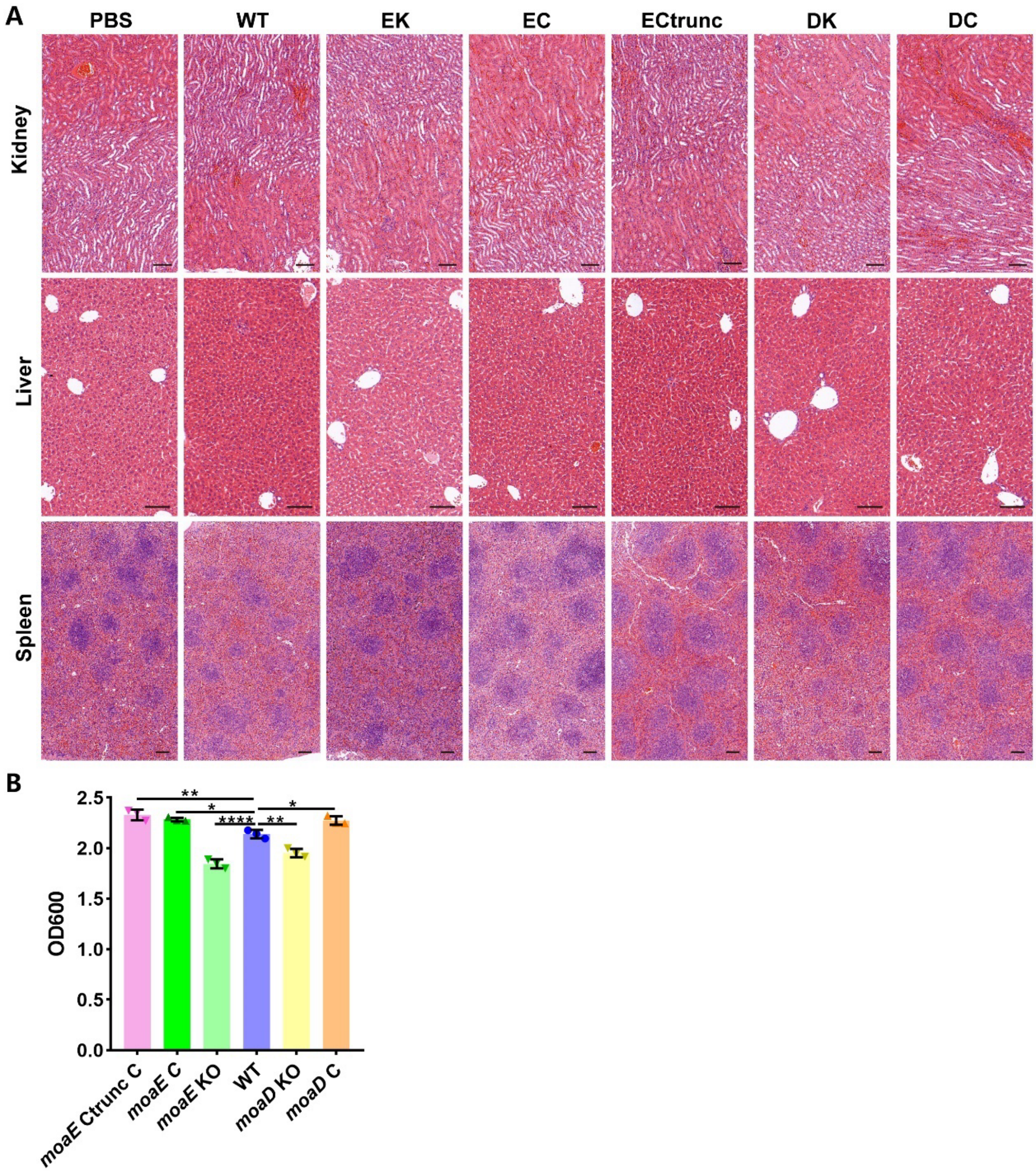

Fig. S6. MPT synthase knock-out in *A. baumannii* ATCC 19606 leads to no obvious damage to kidney, liver and spleen in mice pneumonia model.

(A) Histopathological analysis of kidney, liver, and spleen tissues by Hematoxylin-Eosin staining (HE) staining after WT, DK, DC, EK, EC, ECtrunc and PBS infection mice for 1 day. All images are displayed with *Bars*= 100 μm. (B) OD600 of 24 h cultured strains in LB at 37 ℃, from OD600=0.01 diluted by overnight cultured bacteria. Statistic assay was performed by one-way ANOVA via GraphPad Prism, with four asterisks indicating $P < 0.0001$, three asterisks for $P < 0.001$, two asterisks for $P < 0.01$, and one asterisk for $P < 0.05$.

Table S1. *moa* operon and its upstream and downstream in *A. baumannii* ATCC 19606.

Gene ID	Gene name	Size	Start	End	Strand
GO593_RS17840	*tusE*	2547	3732190	3732501	+
GO593_RS17845	*17845*	1623	3732506	3733357	+
GO593_RS17850	*17850*	2781	3733456	3734895	+
GO593_RS17855	*17855*	630	3734892	3735374	+
GO593_RS17860	*moeA*	2274	3735583	3736818	+
GO593_RS17865	*moaC*	1041	3736818	3737741	+
GO593_RS17870	*moaE*	255	3737765	3738253	+
GO593_RS17875	*moaD*	486	3738257	3738511	+
GO593_RS17880	*moaA*	924	3738512	3739552	+
GO593_RS17885	*17885*	1236	3739779	3742051	+
GO593_RS17890	*mobA*	483	3742202	3742831	+
GO593_RS17895	*17895*	1404	3742828	3745608	+
GO593_RS17900	*nirB*	864	3745627	3747249	+
GO593_RS17905	*nirB*	312	3747262	3749808	+

Table S2. Vectors, strains and primers used in this paper.

**1. Vectors**	
pET28a	pMMB67EH
**2. Strains**	
E. coli BL21 Star^{TM} (DE3), Thermofisher, Cat#: C601003	
Acinetobacter baumannii, ATCC, ATCC19606	
**3. Primers used in this paper**	
***Ab*MoaD pET28a construction**	
F-*Ab*MoaD-OP-28a	TAACTTTAAGAAGGAGATATACCATGCATCATCATCATCATCACATGCAACAATCTATCCAAATTAAAG
R-*Ab*MoaD-OP-28a	GCTTTGTTAGCAGCCGGATCTCAGTGTCATCCTCCGGCAACG
***Ab*MoaE pET28a construction**	
F-*Ab*MoaE-OP-28a	TAACTTTAAGAAGGAGATATACCATGCATCATCATCATCATCACATGAAAGAGTTCGCAAGAATACAAG
R-*Ab*MoaE-OP-28a	GCTTTGTTAGCAGCCGGATCTCAGTGTCAGTGTGAGTGCTCATGGC
***Ab*MoaD knock-out strains of *Ab*19606**	
F-*Ab*MoaD-up	ATCAAATCTTACCTATGGTGAAATG
R-Kan-*Ab*MoaD-up	GAAGCAGCTCCAGCCTACACTTATCCCCCAAGCATATGCA
F-Kan-*Ab*MoaD-down	GGACCATGGCTAATTCCCATGTCATGAAAGA

	GTTCGCAAGA
R-*Ab*MoaD-down	TATTACATATTTCAGTGTGAGTGCT
F-pET28a-*Ab*MoaD-up	GCTTTGTTAGCAGCCGGATCTCAGTGATCAAATCTTACCTATGGTGAAATG
R-pET28a-*Ab*MoaD-down-	GTTTAACTTTAAGAAGGAGATATACCATGTATTACATATTTCAGTGTGAGTGCT
R-ID-AbMoaD-54℃	GTCTTAATAGTTCAATACAGTGC
***Ab*MoaE knock-out strains of *Ab*19606**	
F-*Ab*MoaE-up	GTGACCGTATACGGCTTACCG
R-Kan-*Ab*MoaE-up	GAAGCAGCTCCAGCCTACACGACTCATCCTCCGGCAACG
F-Kan-*Ab*MoaE-down	GGACCATGGCTAATTCCCATAATATGTAATACTAGGATAAATAATGAAAAAC
R-*Ab*MoaE-down	TACGGTATCTGACAATACAATAACC
F-pET28a-*Ab*MoaE-up-	GCTTTGTTAGCAGCCGGATCTCAGTGGTGACCGTATACGGCTTACCG
R-pET28a-*Ab*MoaE-down-	GTTTAACTTTAAGAAGGAGATATACCATGTACGGTATCTGACAATACAATAACC
F-ID-*Ab*MoaE-61℃	CAGCAACATGAGCCAGC
***Ab*MoaD pMMB67EH construction**	
F-pMMB-*Ab*MoaD-Op	CGGATAACAATTTCACACAGGAAACATGCAACAATCTATCCAAATTAAAG
R-pMMB-*Ab*MoaD-Op	CTTCTCTCATCCGCCAAAACAGTCATCCTCCGGCAACG
***Ab*MoaE pMMB67EH construction**	
F-pMMB-*Ab*MoaE-Op	CGGATAACAATTTCACACAGGAAACATGAAAGAGTTCGCAAGAATACAAG
R-pMMB-*Ab*MoaE-Op	CTTCTCTCATCCGCCAAAACAGTCAGTGTGAGTGCTCATGGC
***Ab*MoaE ctrunc pET28a/pMMB67EH construction**	
F-ECtrunc-tga mutant-66.1℃	TTCGTAAGtgaGCACCGCACGACCACAAACAT
F-ECtrunc-tga mutant-60.2℃	CGGTGCtcaCTTACGAATACAACAGCCCTCTACA
***q*RT-PCR**	
F-16srRNA-q	CAGCTCGTGTCGTGAGATGT
R-16srRNA-q	CGTAAGGGCCATGATGACTT
F-*moaD*-q	AAAATTGCTTCCTGAAAATC
R-*moaD*-q	AAGTGCATGGCTCACTTAA
F-*moaE*-q	GAAGGTAAAGCGGTAAAGG
R-*moaE*-q	GAACCACTCGCACATAAGA
F-*bfmR*-q	CTTGGTCTGGAAATGGGTG
R-*bfmR*-q	TGACAAGGTCGTCAAACTC

F-*katE*-q	CTCGCCATTACTTACTCGA
R-*katE*-q	ATGTGCGGCTATTTGTTCT
F-*bfr*-q	CGTGGCAATCCAGAAGTCA
R-*bfr*-q	ATAATCGCATCGGCGTGAG
F-*katG*-q	CACTAACTGGGATGGTTCTC
R-*katG*-q	TGCCTGATTTACGGTCTTT
F-*mgtA*-q	CCAATGCTGCCTATTCACT
R-*mgtA*-q	GCTAATCGGTCCAAACACC
F-*merR*-q	TTCGTGAAATCGGCACAGC
R-*merR*-q	GCCAAGTCGGCCATCTTCT
F-*maiA*-q	AAGGCTTTCAAACACTCGA
R-*maiA*-q	AGAAGGCTGGGATAGACAA
F-*bauB*-q	CCCGACCATTCATTACGAT
R-*bauB*-q	ATGCTCTTTCTGGGCGATT
F-*basG*-q	ATCGCTTTCAGGTAGTGGG
R-*basG*-q	TGTATCTCGGTGATGTGCC
F-*basB*-q	GGGGCAACCAAAGGTCTAAG
R-*basB*-q	TGCATGGGCGATAGCAGTA
F-*basF*-q	ACCCTTTCTGGTTGGAGAT
R-*basF*-q	AGGTCGTGTAATGCCTGTT
F-*gabT*-q	GTGGTGTAGGCGTGATGTG
R-*gabT*-q	TGGCGTAACTTGATAAGCAGT
F-*trmB*-q	CGTTTATGCGTCGTTCAGC
R-*trmB*-q	TAGTTGCGTTCAGGGTTCG
F-*cydB*-q	ATTAAGAATAGCCAAGAACG
R-*cydB*-q	AAATTATAGGCAGGTAGAGC
F-*betB*-q	TTCACGGGTGGCGTAGAAA
R-*betB*-q	GGCACGGTTAAGGTCAGCA
F-*betI*-q	ACCGAACAGTACAGAGCAG
R-*betI*-q	GTTGGAATACAGGCGTTGA

Table S3. X-ray data collection and refinement statistics.

Crystal	MoaDE Complex
**Data collection**	
Spacegroup	$P4_12_12$
a, b, c (Å)	109.4, 109.4, 121.4
α, β, γ (°)	90.0, 90.0, 90.0
Resolution (Å)	40.65-1.97 (2.04-1.97)
*R*merge	0.061 (1.258)
*R*meas	0.062 (1.283)
Multiplicity	25.8 (26.0)
CC (1/2)	1 (0.932)
CC*	1 (0.982)
I/σ(I)	36.63 (2.65)
Completeness (%)	94.63 (99.54)
Wilson B-factor ($Å^2$)	41.16
**Refinement**	
Total Reflections	1298617 (135957)
Unique Reflections	50348 (5233)
R_{work}	0.1930 (0.2494)
R_{free}	0.2210 (0.2969)
Number of atoms:	
Macromolecules	3527
Water	256
Ligand	42
Average B-factor ($Å^2$)	47.76
Protein ($Å^2$)	47.30
Water ($Å^2$)	51.29
Ramachandran plot:	
Favored/Allowed (%)	98.65/1.35
Root-Mean-Square-Deviation:	
Bond lengths (Å)	0.008
Bond Angle (°)	0.95

Statistics for the highest resolution shell are shown in parentheses.

Table S4. Summary of hydrogen bonds and salt bridges of interaction amino acids between MoaE.

Number	MoaE	MoaE
**Hydrogen bonds**		
**1**	Asn 38 [N]	Pro 24 [O]
**2**	Thr 35 [OG1]	Cys 26 [O]
**3**	Thr 35 [N]	Gly 27 [O]
**4**	Met 33[N]	Ile 29 [O]
**5**	Ile 31 [N]	Ile 31 [O]
**6**	Ile 29 [N]	Met 33 [O]
**7**	Gln 21 [NE2]	Gly 89 [O]
**8**	Arg 102 [NE]	Glu 110 [OE2]
**9**	Arg 102 [NE2]	Glu 114 [OE2]
**10**	Pro 24 [O]	Asn 38 [N]
**11**	Cys 26 [O]	Thr 35 [OG1]
**12**	Gly 27 [O]	Thr 35 [N]
**13**	Ile 29 [O]	Met 33 [N]
**14**	Ile 31 [O]	Ile 31 [N]
**15**	Met 33 [O]	Ile 29 [N]
**16**	Thr 35 [OG1]	Gly 28 [N]
**17**	Glu 110 [OE2]	Arg 102 [NE]
**Salt bridges**		
**1**	Arg 102 [NE]	Glu 110 [OE2]
**2**	Arg 102 [NH2]	Glu 114 [OE2]
**3**	Glu 110 [OE2]	Arg 102 [NE]
**4**	Glu 114 [OE2]	Arg 102 [NH2]

Table S5. Summary of hydrogen bonds and salt bridges of interaction amino acids between MoaE and MoaD.

Number	MoaE	MoaD
**Hydrogen bonds**		
**1**	Tyr 135 [OH]	Glu 16 [OE1]
**2**	Arg 142 [NH1]	Glu 52 [O]
**3**	Arg 142 [NH1]	Glu 52 [OE1]
**4**	Cys 140 [SG]	Lys 53 [O]
**5**	Tyr 53 [OH]	Gly 59 [O]
**6**	Lys 59 [NZ]	Asn 61 [OD1]
**7**	Cys 139 [N]	Val 81 [O]
**8**	Tyr 50 [OH]	Gly 84 [OXT]
**9**	Lys 124 [NZ]	Gly 84[OXT]
**10**	Cys 139 [O]	Val 81 [N]
**11**	Val 122 [O]	Ala 82 [N]
**12**	Lys 117 [O]	Gly 83 [N]
**13**	Val 122 [O]	Gly 83 [N]
**Salt bridges**		
**1**	Arg 142 [NH1]	Glu 52 [OE1]
**2**	Lys 124 [NZ]	Gly 84 [O]
**3**	Lys 124 [NZ]	Gly 84 [OXT]
**4**	Glu 125 [OE1]	Lys 17 [NZ]
**5**	Glu 125 [OE2]	Lys 17 [NZ]

Table S6 Summary of top6 Dali search against PDB50 of chain C (MoaD) and chain D (MoaE) in MPT synthase.

**MoaD**								
**Number**			**Chain**	**Z**	**RMSD**	**Lali**	**Nres**	**% id**
**1**	*Mycobacterium tuberculosis*	*Mt*MoaD	6jbz-B	13.5	1.5	82	84	22
**2**	*Homo sapiens*	*Hs*MoaD	5mpo-B	11.6	1.7	80	84	15
**3**	*Haloferax volcanii*	*Hv*MoaD	3po0-A	11.1	2.0	80	89	18
**4**	*Pyrococcus furiosus*	*Pf*MoaD	1vjk-A	11.1	1.6	76	87	20
**5**	*Staphylococcus aureus*	*Sa*MoaD	2q5w-D	10.7	1.7	76	77	22
**6**	*Thermus*	*Tt*MoaD	1v8c-A	9.6	1.7	74	165	27

	thermophilus							
**MoaE**								
**1**	*Staphylococcus aureus*	*Sa*MoaE	2q5w-E	19.2	1.8	134	137	30
**2**	*Deinococcus radiodurans*	*Dr*MoaE	8hlg-A	18.0	1.9	131	137	33
**3**	*Mycobacterium tuberculosis*	*Mt*MoaE	6jbz-A	17.9	1.6	130	141	35
**4**	*Aquifex aeolicus*	*Aa*MoaE	2omd-B	17.6	2.1	131	145	31
**5**	*Homo sapiens*	*Hs*MoaE	4ap8-A	17.3	1.6	123	134	37
**6**	*Escherichia coli*	*Ec*MoaE	1fm0-E	15.7	2.2	127	142	28

Table S7 SAXS data collection and analysis statistics.

Samples	$(MoaD\text{-}MoaE)_2$
**Data Collection Parameters**	
Beamline	BL19U2, SSRF, Shanghai
Beam size	0.33mm×0.55mm
Detector	Pilatus 1M
q range (Å^{-1})	0.04-0.45
Temperature	293K
Concentration	5mg/mL
**Molecular Weight Determination**	
Sequence weight (kDa)	58.4
SAXS MoW2 (kDa)	55.9
Porod Volume (Vp/1.7)	50.1
